# Optimized deep residual networks for early detection of myocardial infarction from ECG signals

**DOI:** 10.1186/s12872-025-04739-z

**Published:** 2025-05-17

**Authors:** Pon Bharathi A, Madavan R, Sakthivel E

**Affiliations:** 1 Department of Electronics and Communication Engineering, Amrita College of Engineering and Technology, Nagercoil, Kanyakumari, Tamil Nadu 629901 India; 2 Department of Electrical and Electronics Engineering, K. Ramakrishnan College of Technology, Trichy, Tamil Nadu India; 3 Department of Electrical and Electronics Engineering, PSRR College of Engineering, Sivakasi, Tamil Nadu 626140 India

**Keywords:** MI, Deep Learning, ECG, Optimization, SSS-DRN, Feature Extraction, Data Augmentation

## Abstract

Globally, the high number of deaths are happening due to Myocardial infarction (MI). MI is considered as a life-threatening disease, which leads to an increase number of deaths or damage to the heart, and hence, prompt detection of MI is critical to decrease the mortality rate. Though, numerous works have addressed MI identification, an increased number suffer from over fitting and high computational burden in real-time scenarios. The proposed system introduces a novel MI detection technique using a Deep Residual Network (DRN), where the solution is optimized by the proposed Social Ski-Spider (SSS) Optimization algorithm is the novel combination of both Social Ski-driver (SSD) Optimization and the Spider Monkey Optimization (SMO). This model highly prevents the overfitting and computational burden, which increases the MI detection accuracy. Here, the proposed SSS-DRN performs detection by filtering the electrocardiography (ECG) signals. Later, the signal feature, transform feature, medical feature and statistical feature are extracted by the feature extraction phase followed by data augmentation that consists of permutation, random generation and re-sampling processes and finally, detection is accomplished by the SSS-DRN. Moreover, the developed SSS-DRN is researched for its efficiency considering metrics like accuracy, sensitivity, and specificity and observed 0.916, 0.921, and 0.926. Here, when considering the accuracy metrics, the performance gain observed by the devised model is 13.96%, 12.61%, 10.37%, 7.95%, 5%, 2.21%, and 2% higher than the traditional schemes. This indicates the devised model has high detection accuracy, which could be embedded in real-time clinical settings like hospital ECG machines, wearable ECG monitors, and mobile health applications. This improves the clinical decision-making process with increased patient outcomes.

## Introduction

Coronary or ischemic heart disease is a major health complexity contributing to an increased mortality rate, as per the World Health Organization (WHO). A key reason for cardiovascular disease among patients is MI, most often addressed as a heart attack [50]. MI is the most commonly found cardiac disease resulting from chronic myocardial ischemia [32]. MI is considered as a life-threatening disease that occurs when the blood retentiveness is increased owing to coronary artery blockage resulting to total death or damage to the heart. This condition is generally a medical emergency that necessitates urgent medical care [24]. For prompt treatment, precise and timely diagnoses of MI are essential. Clinically, MI is most commonly diagnosed using Myocardial Contrast Echocardiography (MCE) but clinical examination based on MCE has a high dependence on the operator and is subjective and laborious. The automated detection of MI based on MCE reduces the burden on medical professionals and helps in improving the efficiency of diagnosis [23]. Numerous signal processing techniques have been devised for identifying MI at the earliest using ECG. By observing variations in the various time-domain parameters that include T-wave inversion, Q-wave amplitude, and ST deviation, the ECG signal can effectively diagnose MI. By utilizing 12 lead ECG recordings, the features are being extracted in order to identify MI. The variations can be employed for diagnosing and localizing the portion of the heart muscle affected and the portion of the coronary artery facing blockage [6]. MI diagnosis and localization are performed by taking the Principal Component Analysis (PCA) of all leads based on the features obtained from the Q-wave of the heartbeat and the ST-T region [5]. MI is detected and localized in the left ventricle, considering the T-wave integral and the overall ECG beat integral [49].

ECG signal is obtained due to the effect of electrical conductivity in the myocardium cells that makes the heart muscles to contract and relax interchangeably in the auricles and ventricles [8]. ECG signals are recorded by keeping the electrodes at pre-determined locations in the human body. The pathological and morphological characteristics of the ECG signals vary during the occurrence of MI. The advancement in digitization empowers the systems the ability to process and acquire information from the ECG signals in digital form [38]. Computer-aided diagnosis (CAD) systems are used for diagnosing cardiac diseases based on the ECG signals, and they are highly robust, precise, fast and reliable in comparison to the traditional approaches employed earlier [47, 49]. Detecting myocardial ischemia as early as possible is a crucial problem as it can minimize the rate of mortality, acute myocardial infarction, and other dangerous cardiovascular problems. Myocardial ischemia can lead to heterogeneity of repolarisation, owing to the variation in ventricular repolarisation caused by electrophysiological changes [31]. Currently, 12-lead ECG is extensively used to monitor the electrical activities in the heart and can effectively trace the abnormal variations in the ST-T segments in ECG caused by the heterogeneity of repolarisation [48].

Recent years have witnessed the emerging growth of Deep Learning (DL) techniques in the field of MI detection because of their highly accurate performance, devoid of any massive signal processing needs [2, 19, 30]. DL approaches, such as Convolutional Neural Networks (CNN), Recurrent Neural Networks (RNN), Restricted Boltzmann Machines (RBM), and Auto Encoder (AE) are utilized by the clinical system for analysing physical signals, like Electroencephalogram (EEG) and ECG [25] [16] [14]. DL is a kind of neural network that executes an automatic ranking of features based on a multi-layer hierarchy. Several studies have addressed the problem of estimating ventricular volume, segmenting, and categorization by combining DL schemes with random forests [23]. However, DL techniques utilize a convolution (conv) filter having a rectangular shape, and the myocardium is ring-shaped, specifically considering the MCE’s short axis view [15]. The usage of a rectangular conv filter limits the ability of the DL to describe the features and information in the radial direction [4, 28]. Several DL and Machine Learning (ML) schemes have been devised to analyze ECG waves for localizing, detecting, and classifying MI, based on the features mined using neural networks, wavelet transform, Fourier transform, Support Vector Machines (SVM), or with the help of Deep Neural Networks (Deep NN), directly [48]. Traditionally, ML methods extract the various features of ECG, like its frequency domain, wavelet transform, and time domain, amplitude. The feature selection process reduces the computational complexity of the ML algorithms as they contain required informative features [33, 34, 39]. The modern ML approaches utilize classifiers, like Deep NN, which extract the features automatically [3, 56]. Though several automatic MI diagnosis systems using various approaches to feature extraction have been developed, the efficacy of the techniques depends on the optimal choice of the extracted features [55].

### Problem statement and motivation

MI arises as a harmful disease and the diagnosis of its occurrence time is essential to provide intervention of disease earlier thereby supporting the patients suffering from cardiovascular disease. Early detection helps in reducing the risk factors and limits the post-complications of this disease-causing heart failure. The complications and heart failure possessed by this disease can be reduced when detecting it earlier. The high mortality rate related to cardiovascular disease is raised due to MI. To detect MI earlier, the DL models are used for providing automatic detection of MI. The problems encountered by the classical models delay the detection. The generalizability of the traditional models is affected due to the lack of diverse and well-annotated datasets. The conventional schemes heavily relied on supervised learning and this requires a huge number of labelled data for tuning the model, which remains a major limitation. When dealing with large datasets in real-world conditions, the traditional model struggles with the computational burden. Also, these models require a vast amount of hardware resources and this delays the detection results, which creates problems in emergency situations. The transfer learning approaches used failed to generate synthetic data, which increases the overfitting issues and biases of the model. The existing models failed to handle the class imbalance issues, which makes the results of the model a biased one. The improper selection of filter and feature extraction variation associated with the traditional models produces inconsistencies in the data thereby reducing the performance of the model. The traditional model failed to address the challenges such as the need for large and varied datasets, possible biases in data collection, and the interpretability of deep learning models. Numerous works have addressed the issue in MI detection and have developed automatic MI classification schemes from ECG signals. Most of the approaches utilize algorithms that have high computational cost and authentication time, and their performance is impacted by the ECG signal quality. In order to overcome these challenges, this research proposed a DL model for the detection of MI. The use of DRN with SSS algorithm improves the performance of the model with improved generalizability thereby mitigating the overfitting and computational issues. Here, the DRN is effective in preventing vanishing gradient problems and has the capability of capturing complex patterns from the ECG data. This model is highly effective in handing large dataset due to the residual connections associated with the DRN. The effective tuning of DRN by the SSS improves the detection process and ensures that the model is computationally efficient. Thus, the integration of DRN with SSS improves the generalizability of the model and makes the model reliable in the real-time clinical environment.

### Contribution

This paper focuses on developing an enhanced MI detection scheme using SSS-DRN. The novel contribution is stated below,

- Proposed SSS-DRN for MI detection: This paper presents a novel MI detection approach using SSS-DRN, wherein DRN is used to identify MI from the ECG signals. The proposed SSS algorithm is formulated using SMO and SSD, which improves the detection process.

### Organization

- The organization of the work is presented here; an elaborated view of the related work is elucidated in second section, the developed SSS-DRN for MI identification is focused in third section, Fourth section presents the experimental outcomes, evaluation of the technique based on the outcomes, and is concluded in the fifth section.

## Literature review

Numerous DL schemes have effectively accomplished the MI detection task; in this section, a few methods are contemplated for assessment. Alghamdi et al. [1] presented a CNN-based approach for identifying MI, where two classes of transfer learning schemes are used. Here, two networks were developed, such as Visual Geometry Group-MI1 by performing the fine-tuning VGG-Net and VGG-MI2 for extracting features. This technique did not require additional feature extraction and segmentation techniques to achieve enhanced accuracy,however, the approach did not consider testing more data to augment the system efficiency. Swain et al. [49] proposed a Modified Stockwell transform (MST) and Phase distribution pattern to recognize the MI occurrences from the ECG signals. Here, MI was detected by considering the phase distribution pattern of Health Control (HC) and MI ECG signals. To determine the phase details of the ECG signals, the MST was employed and this information was used in order to identify MI. This technique offered high performance without needing any past information on MI but suffered from higher computational costs. Lin et al. [36] developed a k-Nearest Neighbour (kNN) approach for classifying MI. This scheme calculated five types of features, covering information, time-series similarity, and energy to examine the MI and HC ECG signals. Moreover, user-specified thresholds and the Student’s test are implemented to choose the best feature set in the feature selection process. Finally, kNN classified the signals based on the feature set obtained. This approach effectively overcame the issues arising due to outliers, but it required the extraction of numerous features, which lead to high computational complexity [30] . introduced a Shallow and End-to-End Deep NN for localizing and detecting MI. The features were directly extracted by the Shallow and End-to-End Deep NN technique from the pre-processed signals using the CNN and the generated feature was employed using Deep NN. This approach offered high classification accuracy without the need for any additional processing of signals but failed to work effectively with signals affected by noise. Sun et al. [48] presented a Lempel–Ziv (LZ) Complexity based technique for detecting Myocardial Ischemia. Myocardial ischemia was detected using the LZ and Lyapunov exponent (LYE) models constructed by combining the Fourier transform coefficient with the LZ complexity and LYE obtained from the CDG. This method had low computational complexity,however, this method considered only fewer samples and hence was unsuccessful in verifying whether quantifying CDG is sufficient for detecting MI. Sharma and Sunkaria [46] introduced an Optimal Features Based Lead Specific Approach for classifying MI. This approach used stationary wavelet transform to decompose the processed signals to wavelet bands, from which slope, entropy, and energy-based features were computed. The classification was performed using the kNN. This approach offered high accuracy even when the signal was acquired from a single lead but was not generalizable owing to the lack of utilization of a large database in validation. Han and Shi [25] devised a Multi-lead residual neural network (ML-Res Net) to locate and identify MI. Here, the features were captured from the ECG signals by utilizing 3 residual blocks. A feature fusion technique was employed to localize and identify MI from the 12 lead ECG signals. This scheme effectively produced highly accurate results,however, it suffered from poor performance in localizing MI in the inter-patient method. Liu et al. [37] introduced a Dual-Q Tunable Q-factor Wavelet Transformation (Dual-Q TQWT) and wavelet packet tensor decomposition technique to detect and locate MI. The de-noised and segmented ECG signals were subjected to Discrete Wavelet Packet Transform (DWPT) for constructing a fourth-order wavelet tensor for representing the various features of the ECG signal. Later, Multi linear Principal Component Analysis (MPCA) was utilized to protect the intrinsic details and minimize the dimensions of the tensor. Finally, classification was accomplished using bootstrap aggregated decision trees (Tree bagger) classifier. Though this technique was highly robust, it was unsuitable for detecting other heart diseases. Hao et al. [26] devised a MI detection framework using multi-branch fusion framework. Here, features extracted using a multi-branch network from the 12 leads were subjected to a feature fusion module to integrate the obtained information, and classification was performed to identify MI. This approach effectively detected MI at high speed,however, it failed to produce accurate results in the case of ECG images with unclear or missing texts. Guo et al. [23] proposed a Polar Residual Network (PResNet) to localize MI based on the MCE images. This technique devised a polar region to consider the myocardium’s ring shape. MCE images were subdivided into various sections and applied to the Pres Net, where the images were classified. This method enhanced the prominent features, thereby providing efficient classification, although the technique endured high computational costs. Deepika and Jaisankar [13] presented a technique, named CNN and an Echo Cardiogram Video (ECV- 3D) network for detecting and classifying the MI from echocardiogram frames. However, the ECV- 3D had a few issues interpretability challenges, training requirements, initial investment and data dependence in ensuring the algorithm’s implementation. Golande and Pavankumar [22] presented a hybrid filtering technique by considering CNN-based features and classifying them using the Long Term Short Memory (LSTM) classifier. However, this model was not effective in detecting MI from raw ECG signals. Bender et al. [9] introduced a quantitative analysis pipeline technique by using Deep Neural Networks (DNN) and applied Explainable Artificial Intelligence (XAI) methods using public ECG databases. Though the pipeline technique had high effectiveness, the public ECG databases introduced certain biases that results in improper results during the emergency situation. Safdar et al. [43] presented a Data Augmentation (DA) approach to enhance an ECG dataset of samples from ECG signals. However, the performance of the model is not satisfactory for other types of diseases like long ST intervals or long pauses between two cardiac cycles.

### Challenges

A few issues that were met during the detection of MI are listed as follows.

- The CNN-based technique in [1] for the detection of MI is that the approach did not utilize data augmentation schemes to increase the efficiency of the developed technique. Further, the approach wasn’t extended to identify various kinds of cardiac disorders, like Atrial Flutter (AF), Ventricular fibrillation (V-Fib), and Atrial fibrillation (A-Fib).
- A kNN classifier was developed in [36] for identifying MI and this technique utilized a simple feature extraction technique, which was effective in increasing the speed of detection. However, this approach was unsuccessful in analyzing the features extracted for their distinct clinical significance, to enhance the efficiency of identification.
- In [30], Deep NN was developed for localizing and detecting MI with high accuracy, however, no hardware implementation was considered for detecting MI proactively. Further, it failed to consider real-time detection by modifying the standard ECG machine with an additional electronic circuit.
- The ML-Res Net was presented in [25] for identifying and localizing MI and excellent results with inter-patient schemes. However, the limited number of patient data limits the efficacy of MI detection in intra-patient methods, which remained a key challenge.
- The existing MI detection techniques do not provide high accurate results in the presence of the noises in ECG signals due to muscle contraction, power line interference, and Baseline Wanders (BW) that affect the stability of the detection schemes. Further, developing effective feature extraction techniques with minimal complexity is challenging.

## Proposed social ski-spider optimization algorithm for myocardial infarction using a deep residual network

**Fig. 1:**
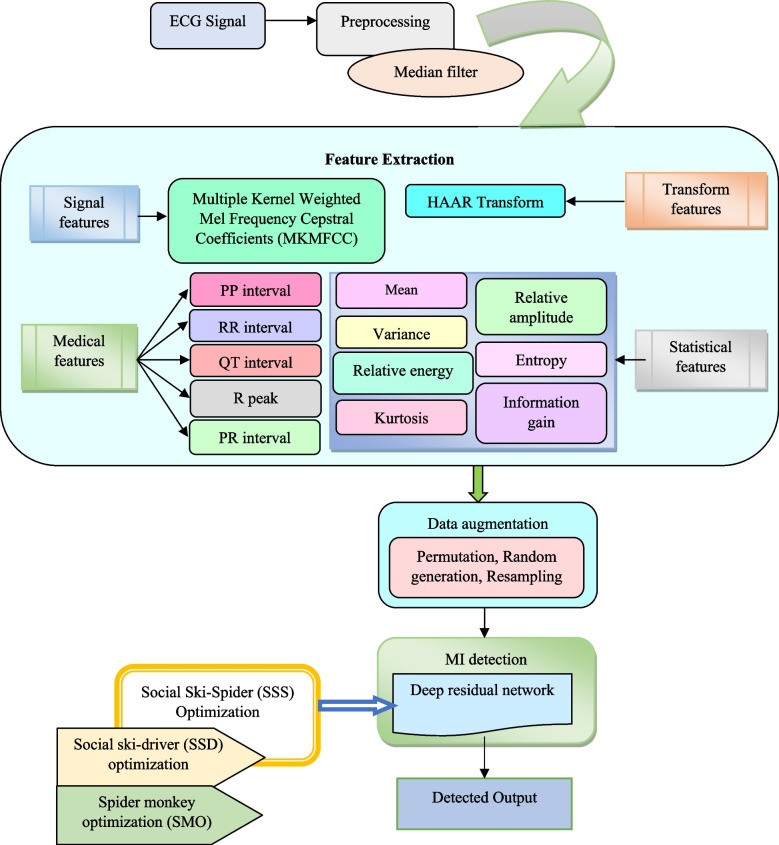
Structural design of developed SSS-DRN for MI detection

In this paper, the SSS-DRN technique is used for detecting MI, which is elaborated in this section. Signal processing is the first step being employed in this technique; wherein the noises in the ECG signal is removed using the median filter [35]. This is followed by the identification of the most discriminative features from the noise-free ECG signals. Features, like Multiple Kernel Weighted Mel Frequency Cepstral Coefficients (MKMFCC) [18], medical features, including R peak, QT interval, RR interval, PR interval, and PP interval, transform features like HAAR transform [12], and the statistical features such as mean, variance, relative energy, relative amplitude, entropy, kurtosis, information gain are identified. After extracting the salient features in ECG signals, it is followed by data augmentation. It produces more information from the real ECG signals for overcoming the possibility of inaccurate prediction and over fitting arising due to the minimal ECG samples. By using permutation, random generation and re-sampling, data augmentation is being performed. Finally, MI identification is performed using the DRN [11], whose weight parameters are tuned based on the devised SSS algorithm. The SSS algorithm is formulated by adapting the SSD [52] algorithm based on the SMO [7] algorithm. Figure 1 shows the schematic view of the devised SSS-DRN model for detecting MI.

### ECG signal acquisition

The process of MI detection is established by considering the dataset $K$, which contains a total of $k$ ECG signals, that is represented by,

1 $$K = \left\{ {K_{1} ,K_{2} ,..,K_{i} ,..,K_{k} } \right\}$$

wherein, $K_{i}$ symbolizes the $i^{th}$ ECG signal, which is the input acquired for further processing and $K_{k}$ is the total samples present in the dataset.

### Median filter for pre-processing

Here, the median filter is used in order to de-noise the acquired ECG signal $K_{i}$[35]. The median filter is a non-linear digital filter that runs the signal depending on each value. The percussive events and harmonic components are suppressed by the median filter and they are separated in horizontal and vertical directions. The output obtained is given by,

2 $$M_{i} \left( {ts} \right) = median\left\{ {K_{i} \left( {ts - ns:ts + ns} \right),ns = {{\left( {len - 1} \right)} \mathord{\left/ {\vphantom {{\left( {len - 1} \right)} 2}} \right. \kern-0pt} 2}} \right\}$$

Here, $len$ represents the length of the samples in the ECG signal. The median filter replaces every value with the middle value of the samples when $len$ is odd, else, it is replaced by the mean of the middle two values. The median filter effectively minimizes the impulse noise in the ECG signals, and the de-noised signal $M_{i}$ is subjected to the feature extraction phase for further processes.

### Extraction of features

The feature extraction phase identifies the most discriminative features from the noise-free ECG signals that is the de-noised signal $M_{i}$ obtained from the previous process. The de-noised signal $M_{i}$ is the input for the feature extraction phase to disclose the hidden characteristics of the input ECG signals. This process extracts the signal feature (MKMFCC) [18], transform feature (HAAR), medical features, including R peak, QT interval, RR interval, PR interval, and PP interval, and statistical features such as mean, variance, relative energy, relative amplitude, entropy, kurtosis, and information gain are identified. These features are elucidated below.

### Medical features

The medical features [10]in the ECG signal are determined by finding the wave components in the ECG signal. The ECG signal comprises various waves, like P wave, T wave, and QRS complex wave. The components in these waves have to be identified for the effective identification of MI. Initially, the wavelet coefficient are chosen appropriately for detecting R-peak and the selection of Q and S points are done based on five-point differentiation strategy. Thereafter, the analysis between the wavelet coefficients of T and S marks is done. The medical features, like R peak, QT interval, RR interval, PR interval, as well as PP interval are computed, which are detailed below.

i) R peak
  It denotes the maximal amplitude of the R wave equalized to create the ECG signal’s R peak aspect, and is obtained as,

3 $$R^{i} = \frac{1}{a}\sum\limits_{b = 1}^{a} {R^{b} }$$

Here, $a$ symbolizes the total number of points in the ECG signal of the $i^{th}$ individual and $R^{b}$ symbolizes the R wave of the $b^{th}$ point.

ii) QT interval
  This feature represents the interval of time amid the among Q and T waves of the $i^{th}$ individual’s ECG, and the mean of all the computed QT intervals is used for finding the QT feature, which is calculated as,

4 $$QT^{i} = \left\{ {QT_{b} } \right.\,;\,\,1 \le b \le a$$

5 $$QT = \frac{1}{a}\sum\limits_{b = 1}^{a} {\left[ {Q_{i}^{b} - T_{i}^{b + 1} } \right]}$$

Here, $Q_{i}^{b}$ and $T_{i}^{b + 1}$ signifies the Q and T waves of the $b^{th}$ and $\left( {b + 1} \right)^{th}$ points, respectively of the $i^{th}$ individual.

iii) RR interval
  It denotes the duration of two subsequent R waves contained in the ECG signals of the $i^{th}$ individual, and the mean of the RR intervals of all the $b$ points is calculated to determine the RR feature, which is given by,

6 $$RR^{i} = \left\{ {RR_{b} } \right.\,;\,\,1 \le b \le a$$

7 $$RR = \frac{1}{a}\sum\limits_{b = 1}^{a} {\left[ {R_{i}^{b} - R_{i}^{b + 1} } \right]}$$

wherein, $R_{i}^{b}$ and $R_{i}^{b + 1}$ signifies the R wave at the $b^{{^{th} }}$ and $\left( {b + 1} \right)^{th}$ points of the $i^{th}$ individual.

iv) PR interval
  PR interval refers to the time duration among the P wave at $b^{th}$ point with the R wave at $\left( {b + 1} \right)^{th}$ point is called as PR interval. This features is obtained using the mean of PR interval at every points of the ECG signal, which is formulated as,

8 $$PR^{i} = \left\{ {PR_{b} } \right.\,;\,\,1 \le b \le a$$

9 $$PR = \frac{1}{a}\sum\limits_{b = 1}^{a} {\left[ {P_{i}^{b} - R_{i}^{b + 1} } \right]}$$

Here, $P_{i}^{b}$ symbolizes the $P$ wave at the $b^{th}$ point of the $i^{th}$ individual.

v) PP interval 
  It refers to the time duration among two subsequent P waves contained in the ECG signal and the PP feature can be obtained by taking the mean of the PP intervals based on every points of the ECG signal, which is expressed a

10 $$PP^{i} = \left\{ {PP_{b} } \right.\,;\,\,1 \le b \le a$$

11 $$PP = \frac{1}{a}\sum\limits_{b = 1}^{a} {\left[ {P_{i}^{b} - P_{i}^{b + 1} } \right]}$$

Here, $P_{i}^{b}$ and $P_{i}^{b + 1}$ signifies the P wave at the $b^{{^{th} }}$ and $\left( {b + 1} \right)^{th}$ points of the $i^{th}$ individual.

The R peak, QT interval, RR interval, PR interval, and PP interval features have a dimension of $1 \times 1$, and are combined to get the medical feature with respect to the $b$ points in the $i^{th}$ individual’s ECG signal, and is denoted by,

12 $$A_{1}^{i} = \left\{ {R^{i} ,QT^{i} ,RR^{i} ,PR^{d} ,PP^{i} } \right\}$$

Here, $A_{1}^{i}$ signifies the feature vector obtained from the $i^{th}$ individual’s ECG signal, and has a size of $\left[ {1 \times 5} \right]$.

### MKMFCC

This feature [18] is highly effective in identifying the significant information in the ECG even in a degraded and noisy environment. It utilizes two kinds of kernel functions for the Mel-frequency cepstral coefficient (MFCC) coefficient weighting. The process of extracting the MKMFCC feature is detailed as follows.

i) Pre-emphasis: This process minimizes low-frequency band amplitude and maximizes the high-frequency band amplitude, which is used for flattening the ECG signal and is expressed as,

13 $$Z(c) = M_{i} (c) - Y*M_{i} (c - 1)$$

Here,$Y$ is a constant, $Z$ stipulates the output signal, $M_{i}$ represents the filtered ECG signals, and $c$ is the ECG sample.

ii) Framing: Here, the filtered ECG signal is subdivided into small $B$ blocks of $C$ samples, wherein each block has a length of 20–40 ms.
iii) Hamming Windowing: This process integrates all the nearby frequencies of the ECG signal, and this is formulated as,

14 $$Z(c) = M_{i} (c)*\omega (c)$$

wherein $\omega \left( c \right)$ represents the hamming window, formulated as,

15 $$\omega \left( c \right) = 0.56 - 0.46\left( {\frac{2\pi c}{{C - 1}}} \right)\,\,\,;0 \le c \le C - 1$$

iv) *Fast Fourier Transform (FFT):* The ECG signal is subjected to FFT in this phase, and the obtained block power spectrum is expressed as,

16 $$D_{z} (f) = \frac{1}{C}\left| {M_{i}^{z} (f)} \right|^{2}$$

Further, the Discrete Fourier Transform (DFT) of the specific block is computed as,

17 $$M_{i}^{z} (f) = \sum\limits_{s = 1}^{C} {Z(c).e^{ - j2\pi cf} } ;\,1 \le f \le F$$

wherein, $f$ specifies the DFT length.

v) Mel filter bank application: Here, the weight sum of the filter spectral components and the output border of the Mel scale filter bank are approximated for removing the signal frequencies, which is done by employing the triangular filter. The following expression stipulates the Mel filter bank,

18 $$D(r) = {{\left( {cFFT + 1} \right) \times q(r)} \mathord{\left/ {\vphantom {{\left( {cFFT + 1} \right) \times q(r)} {Sample\,rate}}} \right. \kern-0pt} {Sample\,rate}}$$

Here, $r$ indicates the $r^{th}$ frame of the ECG signal.

vi) Filter bank Energy: Poer spectrum bonds the filter bank and the values are added up to a few coefficients. The following expression gives the energy of the filter bank.

19 $$\varepsilon (r) = \sum\limits_{c = 0}^{\frac{c}{2}} {\log \left| {M_{i} (c)} \right|\left| {Z(c)} \right|} \left( {f\frac{2\pi }{C}} \right) \times W_{c}$$

wherein, $W_{c}$ symbolizes the multiple kernel weighted function.

vii) Discrete Cosine Transform (DCT): It converts the log Mel spectrum into a spatial domain.

20 $$\varepsilon (r) = \overline{\varepsilon }(f)$$

where,

21 $$\overline{\varepsilon }(f) = \left\{ {\begin{array}{*{20}c} {\varepsilon (r)} & {,f = f_{r} } \\ o & {,otherwise} \\ \end{array} } \right.$$

After the DCT is applied, the cepstral coefficients are calculated using the following equation,

22 $$\alpha \left( c \right) = \frac{1}{{C^{\prime}}}\sum\limits_{c = 1}^{{C^{\prime} - 1}} {\overline{\varepsilon }(f).e^{{jf\left( {{{2\pi } \mathord{\left/ {\vphantom {{2\pi } {C^{\prime}}}} \right. \kern-0pt} {C^{\prime}}}} \right)c}} }$$

wherein, $\alpha \left( c \right)$ designates the MKMFCC feature.

viii) Compute Spectrum and energy value: To minimize the noises and augment the recognition accuracy, the energy patterns and features are added up with the feature vector.
ix) Cepstral normalization: To normalize cepstral coefficients, the mean of all the coefficients are reduced and categorized with a variance, and the MKMFCC feature, thus obtained has a dimension of $1 \times 30$ and is represented by $A_{2}$.

### Transform features

Most selective features in ECG signals are extracted by using the Haar transform to obtain the transform features. Haar or Discrete Wavelet Transform (DWT) offers the fusion of both temporal and frequency-based data. A simple wavelet square-shaped sequence is considered to form the Haar wavelets, and this is expressed as,

23 $$\beta \left( t \right) = \left\{ {\begin{array}{*{20}c} 1 & {,0 \le t < \frac{t}{2}} & {} \\ { - 1} & {,\frac{t}{2} \le t < 1} & {} \\ 0 & {,otherwise} & {} \\ \end{array} } \right.$$

Here, the mother wavelet function is specified as $\beta \left( t \right)$ and the scaling function is given by,

24 $$\phi \left( t \right) = \left\{ {\begin{array}{*{20}c} 1 & {,0 \le t < 1} \\ 0 & {,otherwise} \\ \end{array} } \right.$$

The transform feature, thus generated is designated as $A_{3}$ with dimension $1 \times 4$.

### Statistical features

Various statistical features [27], such as mean, variance, relative energy [40], relative amplitude [21], entropy [46], kurtosis, and information gain are also extracted from the filtered ECG signal, and these signals are exemplified below.

- Mean: By taking the average of ECG signal, the mean features are calculated and is expressed as,

25 $$A_{4} = \frac{1}{C}\sum\limits_{j = 1}^{C} {M_{i} (c)}$$

Here, $A_{4}$ denotes the mean,$M_{i} \left( c \right)$ represents the filtered ECG signals at the $c^{th}$ sample, the total samples in the ECG signal is represented as $C$. The mean feature has a dimension of.

*Variance:* This feature is used to determine the amount of variation of the ECG signal from the mean value of the signal, and is expressed by,

26 $$A_{5} = \frac{1}{C}\sum\limits_{j = 1}^{C} {\left( {M_{i} (c) - A_{4} } \right)^{2} }$$

The variance has a dimension of $1 \times 1$.

- Relative amplitude: Relative amplitude [21]of the ECG signal is defined as the ratio between the maximum amplitude of the ECG signal in one lead and the maximum amplitude in another lead, and is given by,

27 $$A_{6} = \frac{{\left( {d_{m} - d_{n} } \right)}}{{\left( {d_{m} + d_{n} } \right)}},m \ne n$$

Here, $m,n = 1\,to\,C$, $d_{m}$ and $d_{n}$ signifies the lead power in two leads. Relative amplitude is denoted by $A_{6}$ and has a dimension $1 \times 50$.

- Relative energy: This feature [40] helps in differentiating the noises and ECG data within the signal. This feature is determined by estimating the energy of a band to the overall energy which is given as,

28 $$A_{7} = \frac{{\varepsilon_{j} }}{{\sum\nolimits_{j} {\varepsilon_{j} } }}$$

Here,$\varepsilon_{j}$ indicates the energy level at the $j^{th}$ decomposition level, and $A_{7}$ signifies relative energy and has a dimension $1 \times 100$.

- Entropy: It measures the degree of uncertainty within the signal. It means the data related to complexities in the heart [46]. It is of the dimesion $1 \times 1$ and is computed by,

29 $$A_{8} = \sum\limits_{c = 1}^{C} {\log \left( {M_{i} (c)^{2} } \right)}$$

- Kurtosis: This feature is implemented to measure the peak of the ECG signal, and is denoted by $A_{9}$ with dimension $1 \times 1$.
- Information gain: The amount of information acquired from the ECG signals corresponding to the features is given by information gain, and is specified as $A_{10}$ and has a size of $1 \times 1$.

The features thus attained from the filtered ECG signals are combined to obtain the feature vector, which is represented as,

30 $$A = \left\{ {A_{1} ,A_{2} ,A_{3} ,A_{4} ,A_{5} ,A_{6} ,A_{7} ,A_{8} ,A_{9} ,A_{10} } \right\}$$

Here, $A_{1}$ symbolizes the medical features, $A_{2}$ gives the MKMFCC feature, $A_{3}$ is the transform feature, $A_{4}$ signifies the mean, $A_{5}$ designates the variance, $A_{6}$ denotes the relative amplitude, $A_{7}$ represents the relative energy, $A_{8}$ signifies entropy, $A_{9}$ is kurtosis, and $A_{10}$ signifies the information gain. The feature vector $A$ generated has a size of $290 \times 147$, and is then applied to the next phase.

## Data augmentation

In this process, the feature vector $A$ is augmented to produce more information from the real ECG signals to overcome the possibility of inaccurate prediction and over fitting arising due to the minimal ECG samples. Data augmentation is accomplished with the help of three techniques, such as permutation, random generation, and re-sampling [41], which are elaborated on in the ensuing sections.

a Permutation
  In general, the process of arranging various elements sequentially is known as Permutation [41]. If an orderly set is available, then permutation rearranges the elements. Permutation is the modest manner of randomly perturbing the events based on their temporal location, and it is performed in two manners.

At first, permutation can be applied to the records entirely, by performing splitting all samples into $D$ parts consisting of equal length. Later, the disturbed segments were assembled to produce a new recording of the signal. This procedure must be reiterated $d_{H}$ times, where $d_{H}$ represents the feature that is used to balance multiple classes. Permutation has to be carried out, where the samples are not repeated. Permutation is employed in the second approach using the Window Slicing (WS) technique which is efficient in producing diversities.

b Random generation
  From determining the highest and lowest magnitude, the samples are produced.
c Resampling
  After completing the permutation task, the feature diversity is enhanced, but splicing the ECG signal and the random perturbation may destroy the morphologies and order of the heartbeat. To tackle this issue, a re-sampling approach is used. Re-sampling not only enhances the sample diversity but also preserves the physiological data and balances the sample count among the various classes.

The augmented feature set thus produced is denoted as $$V$$, has a dimension of $$20227 \times 14720227$$$$\times 147$$, and is forwarded to the MI detection module for identification of MI.

### MI detection using presented SSS-DRN

The augmented feature set $V$ generated is fed into the DRN, where the DRN is employed to distinguish MI from the ECG signal. Here, the weight optimization of the DRN is carried out using the proposed SSS algorithm, where the SSS algorithm is formulated by adapting the SSD [52] algorithm based on the SMO [7] algorithm.

### DRN

DRN [11] offers the advantage of performing classification with high accuracy and minimal error. Further, it has the ability to tackle overfitting issues. Generally, increasing the layer count of the classified results in enhanced classification accuracy but this may lead to gradient disappearance, and this is effectually handled with the usage of residual blocks. The network is increased in its depth rather than the width, thereby achieving high training speed. DRN includes numerous layers that include average pooling (AvgPool) layers, convolutional (conv) layers, residual blocks, and linear classifiers. These layers are elaborated below.

i) ***Convolution layer***: To reduce the free parameters in the training process and enhance the performance owing to the local receptive field and weight sharing, the convolution layer is employed. The two-dimensional conv (Conv2 d) layer uses a kernel (series of filters) to process the input in a smaller area using local connectivity. It computes the output by sliding the e^th^ filter over the input matrix and then performing d^th^ product of the input and kernel, and this is mathematically represented as,

31 $$Conv2d\left( Y \right) = \sum\limits_{u = 0}^{h - 1} {\sum\limits_{v = 0}^{h - 1} {L_{u,v} \bullet U_{{\left( {m + u} \right),\left( {n + v} \right)}} } }$$

32 $$Conv1d\left( U \right) = \sum\limits_{p = 0}^{{C_{in} - 1}} {L_{p} } * U$$

Here, $U$ indicates the output of the prior layer, $L$ signifies the $h \times h$ kernel matrix, $m,n$ signifies the input coordinates, $u$ and $v$ represents the location index of the kernel, and $L_{p}$ denotes the kernel dimension of the $p^{th}$ neuron.

ii) ***Pooling layer***: For minimizing the spatial dimensions of the feature map, the pooling layer is responsible and is effective in handling over fitting. The pooling layer is generally injected into the consecutive convolutional layers. Here, the Maxpooling layer is employed to work on all slices and depths of the input feature map. The maxpooling layer offers simplicity in processing and higher efficiency and is employed to choose the maximum input value and minimizes the feature map dimensions.
iii) Activation function: The non-linear and complex features in the input are learned using a non-linear activation function, which also enhances the non-linearity associated with the obtained features. The activation function used here is Rectified linear unit (ReLU), and is represented by the expression given below,

33 $${\text{Re}} LU\left( U \right) = \left\{ \begin{gathered} 0\,\,;\,\,U < 0 \hfill \\ U\,\,;\,\,U \ge 0 \hfill \\ \end{gathered} \right.$$

iv) ***Batch normalization (Batch Norm)***: Generally, in DL approaches the entire training data is separated into small groups called mini-batches and the archetype is trained using the mini-batches to attain a balance among computational burden and convergence. However, the training speed and stability are reduced due to the interior covariate shift occurring due to the mini-batches. Hence, to overcome this problem, Batch Norm is used, which performs several activation adjustments and scaling to overcome the interior covariate shift.
v) ***Residual blocks***: The residual blocks refer to the two conv layers that is connected via a shortcut. It encompasses a direct link between the input and outputs, in case both the inputs and output exhibit the same size. While the sizes are varied, a dimension matching parameter is applied for matching the sizes, which is represented as,

34 $$I = k\left( U \right) + U$$

35 $$I = k\left( U \right) + \lambda_{U} U$$

Here, $I$ indicates the output, and $U$ is the input of the residual block, $\lambda_{U}$ is the dimension matching factor, and $k$ is the mapping function.

vi) ***Linear classifier:*** After the conv layer performs the extraction and reduction of features, classification is performed by the linear classifier, which encompasses a Fully Connected (FC) layer and a soft max function. The output obtained is given by,

36 $$I = \lambda U + \kappa$$

**Fig. 2:**
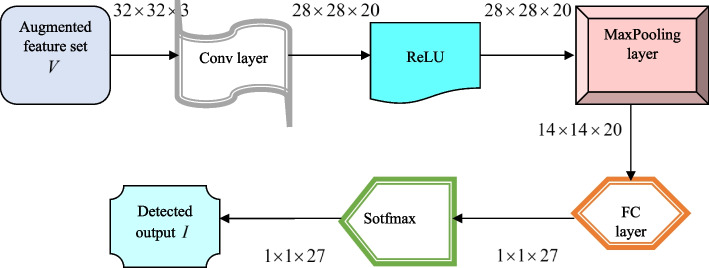
Architecture of DRN

Here, $\kappa$ represents the bias. Figure 2 portrays the architecture of the DRN.

#### Devised SSS algorithm for the weight optimization of the DRN

The efficiency of the DRN in MI identification can be enhanced by using the SSS algorithm in order to adjust the weight parameters of the DRN. Here, the SSS algorithm developed by combining SSD [52] algorithm with SMO [7] algorithm. The SSD algorithm is motivated by the numerous types of evolutionary optimization algorithms and is named based on the stochastic exploration that resembles the paths taken by the ski-drivers downhill. SSD algorithm is based on several parameters, such as agent position, personal best location, mean global best location, and agent velocity. The SMO algorithm, on the other hand, is motivated by the scavenging characteristics of the spider monkeys, which search for food based on fission -fusion characteristics. The spider monkeys divide into numerous groups and search for food in their home region. Here, the group is guided by the female monkey, who also holds the responsibility of identifying the food sources. If the food search is unproductive, the group is further divided into small groups. While food is in surplus, all the groups combine into a large group. Thus, the devised SSS algorithm achieved enhanced exploration and exploitation capabilities from the combination of the SMO and the SSD algorithm. The detailed evaluation steps are given as,

### Step 1: initialization

The primary process is to initialize of the position of the agents, which can be represented as,

37 $$N = \{ N_{1} ,N_{2} , \ldots ,N_{x} , \ldots N_{y} \}$$

wherein, $y$ indicates the overall number of agents and $N_{x}$ represents the position of the $x^{th}$ agent, and all agents are initialized with a corresponding velocity ($O_{x}$).

### Step 2: Error determination

The fitness function of the agents is then calculated, the best solution is obtained by finding the agent with the lowest fitness and hence, the minimization problem is considered. Hence, the fitness is given by Mean Square Error (MSE), which is calculated by,

38 $$Fit = \frac{1}{\rho }\sum\limits_{j = 1}^{\rho } {\left[ {I_{j}^{*} - I_{j} } \right]}^{2}$$

Here, $I_{j}^{*}$ and $I_{j}^{{}}$ signifies the expected and the generated outcome of the DRN, and $\rho$ is the sample count.

### Step 3. Determine the previous best location and the mean global solution

Once the fitness is computed, the agents are sorted based on their fitness, and the previous best location and the mean global solution are determined. The mean global solution is computed using the following equation.

39 $$g_{x}^{k} = \frac{{N_{a} + N_{b} + N_{c} }}{3}$$

Here, $N_{a}$,$N_{b}$ and $N_{c}$ are the three best solutions, and $k$ designates the present iteration.

### Step 4: Determine the update equation

The position of the agents is then modified considering the velocity of the agents, and is given by,

40 $$N_{x}^{k + 1} = N_{x}^{k} + O_{x}^{k}$$

Here,

41 $$N_{x}^{t} = \left\{ \begin{gathered} \begin{array}{*{20}c} {l\sin (o_{1} )(G_{x}^{k} - N_{x}^{k} ) + \sin (o_{1} )(g_{x}^{k} - N_{x}^{k} )\,} & {;if\,o_{2} \le 0.5} \\ \end{array} \hfill \\ \begin{array}{*{20}c} {l\cos (o_{1} )(G_{x}^{k} - N_{x}^{k} ) + \cos (o_{1} )(g_{x}^{k} - N_{x}^{k} )} & {;if\,ot_{2}> 0.5} \\ \end{array} \hfill \\ \end{gathered} \right.$$

wherein, $o_{1}$ and $o_{2}$ are uniformly produced arbitrary number in having values in the range [0, 1], $l$ is the factor that is employed to ensure trade off among exploitation and exploration,$g_{x}^{k}$ represents the best solution of the $x^{th}$ agent.

Considering $o_{2} \le 0.5$, we get,

42 $$O_{x}^{t} = l\sin (o_{1} )(G_{x}^{k} - N_{x}^{k} ) + \sin (o_{1} )(g_{x}^{k} - N_{x}^{k} )\,$$

Applying Eq. (42) in Eq. (40),

43 $$N_{x}^{k + 1} = N_{x}^{k} + l\sin (o_{1} )(G_{x}^{k} - N_{x}^{k} ) + \sin (o_{1} )(g_{x}^{k} - N_{x}^{k} )\,$$

From the SMO algorithm [7],

44 $$Sm_{xw}^{k + 1} = Sm_{xw}^{k} + o\left( {0,1} \right) \times \left( {Gl_{w} - Sm_{xw}^{k} } \right) + o\left( { - 1,1} \right) \times \left( {Sm_{tw}^{k} - Sm_{xw}^{k} } \right)$$

Here, $o(0,1)$ and $o( - 1,1)$ are uniformly produced arbitrary number in having values in the range $[0,1]$ and $[ - 1,1]$ respectively, $Gl_{xw}$ indicates the $w^{th}$ dimension of the global leader location, and $Sm_{xw}^{k}$ and $Sm_{tw}^{k}$ refer to the $w^{th}$ dimension of the $t^{th}$ and $x^{th}$ spider monkey in the $k^{th}$ iteration.

Now, consider $Sm_{xw}^{k + 1} = N_{x}^{k + 1}$, $Sm_{x}^{k} = N_{x}^{k}$, $Gl_{w} = Gl_{t}$, and $Sm_{tw}^{k} = Sm_{t}^{k}$, the Eq. (44) can be rephrased as,

45 $$N_{x}^{k + 1} = N_{x}^{k} + o\left( {0,1} \right) \times \left( {Gl_{t} - N_{x}^{k} } \right) + o\left( { - 1,1} \right) \times \left( {Sm_{t}^{k} - N_{x}^{k} } \right)$$

46 $$N_{x}^{k + 1} = N_{x}^{k} \left[ {1 - o\left( {0,1} \right) - o\left( { - 1,1} \right)} \right] + o\left( {0,1} \right) \times Gl_{t} + o\left( { - 1,1} \right) \times Sm_{t}^{k}$$

47 $$N_{x}^{k} = \frac{{N_{x}^{k + 1} - o\left( {0,1} \right) \times Gl_{t} - o\left( { - 1,1} \right) \times Sm_{t}^{k} }}{{\left[ {1 - o\left( {0,1} \right) - o\left( { - 1,1} \right)} \right]}}$$

Applying Eq. (47) in Eq. (43),

48 $$N_{x}^{k + 1} = \left[ {\frac{{N_{x}^{k + 1} - o\left( {0,1} \right) \times Gl_{t} - o\left( { - 1,1} \right) \times Sm_{t}^{k} }}{{\left[ {1 - o\left( {0,1} \right) - o\left( { - 1,1} \right)} \right]}}} \right] + l\sin (o_{1} )(G_{x}^{k} - N_{x}^{k} ) + \sin (o_{1} )(g_{x}^{k} - N_{x}^{k} )\,$$

49 $$N_{x}^{k + 1} - \frac{{N_{x}^{k + 1} }}{{\left[ {1 - o\left( {0,1} \right) - o\left( { - 1,1} \right)} \right]}} = l\sin (o_{1} )(G_{x}^{k} - N_{x}^{k} ) + \sin (o_{1} )(g_{x}^{k} - N_{x}^{k} )\, - \left[ {\frac{{o\left( {0,1} \right) \times Gl_{t} + o\left( { - 1,1} \right) \times Sm_{t}^{k} }}{{\left[ {1 - o\left( {0,1} \right) - o\left( { - 1,1} \right)} \right]}}} \right]$$

50 $$\frac{{N_{x}^{k + 1} \left[ {1 - o\left( {0,1} \right) - o\left( { - 1,1} \right)} \right] - N_{x}^{k + 1} }}{{\left[ {1 - o\left( {0,1} \right) - o\left( { - 1,1} \right)} \right]}} = \frac{{\left( \begin{gathered} \left( {l\sin (o_{1} )(G_{x}^{k} - N_{x}^{k} ) + \sin (o_{1} )(g_{x}^{k} - N_{x}^{k} )} \right)\,\left[ {1 - o\left( {0,1} \right) - o\left( { - 1,1} \right)} \right] \hfill \\ - \left( {o\left( {0,1} \right) \times Gl_{t} + o\left( { - 1,1} \right) \times Sm_{t}^{k} } \right) \hfill \\ \end{gathered} \right)}}{{\left[ {1 - o\left( {0,1} \right) - o\left( { - 1,1} \right)} \right]}}$$

51 $$N_{x}^{k + 1} = \frac{{\left( {\left( {o\left( {0,1} \right) \times Gl_{t} + o\left( { - 1,1} \right) \times Sm_{t}^{k} } \right) - \left( {l\sin (o_{1} )(G_{x}^{k} - N_{x}^{k} ) + \sin (o_{1} )(g_{x}^{k} - N_{x}^{k} )} \right)\,\left[ {1 - o\left( {0,1} \right) - o\left( { - 1,1} \right)} \right]} \right)}}{{o\left( {0,1} \right) + o\left( { - 1,1} \right)}}$$

Here, $N_{x}^{k + 1}$ gives the position of the $x^{th}$ agent in the next iteration and the above equation is employed for updating the location of the agent.

### Step 5: Feasibility evaluation

After updating the location of the agent, the feasibility of the revealed solution is evaluated by computing the fitness based on Eq. (38), to find the agents with minimal fitness.

### Step 6: Terminate

The above process is reiterated till the stopping criteria are achieved, the pseudo-code of the proposed SSS algorithm is displayed in algorithm 1.

**Figure:**
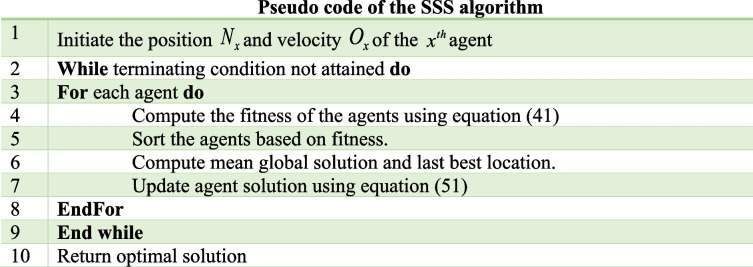
Algorithm 1. Pseudo code of the proposed SSS algorithm

Thus, by combining the SMO, and the SSD algorithm, the devised SSS algorithm achieved enhanced exploration and exploitation capabilities. Further, the weight optimization of the DRN using the devised SSS algorithm effectively enhanced the MI identification process.

## Results and discussion

The experiment and the outcomes obtained during the implementation of the presented SSS-DRN approach for MI detection are elucidated here. Furthermore, the effectiveness of the developed scheme is verified with respect to various metrics, in comparison to the available MI detection schemes.

### Experimental set-up

The presented SSS-DRN approach for MI detection is realized by implementation in a Python environment.

### Dataset description

- Dataset 1: The Physikalisch-Technische Bundesanstalt (PTB) database [42] https://www.physionet.org/content/ptbdb/1.0.0 is employed in this work, which is one of the most extensively utilized datasets in the research addressing MI identification. It comprises 549 records of ECG signals acquired from 290 individuals, having ages between 17 and 87. Every individual is indicated by a total of one to five records, measured using the traditional 12 leads with three Frank lead ECGs. Every signal is digitized at a rate of 1000 samples per second. Out of the 290 subjects, 148 subjects have MI.
- Dataset 2: The MIT-BIH Arrhythmia Database [53] (https://www.kaggle.com/datasets/taejoongyoon/mitbit-arrhythmia-database) contains 48 half-hour excerpts of two-channel ambulatory ECG recordings. Among these 48 recordings, 23 of them were selected randomly from the 4000 24-h ambulatory ECG recording set, which is gathered from the mixed population of inpatients (nearly 60%) and outpatients (40%). The balance of 25 recordings are selected from less common, which include arrhythmias in the minimum random sample.

### Evaluation measures

Based on measures, like accuracy, sensitivity and specificity, the devised SSS-DRN for MI identification undergoes the process of evaluation that is explicated below.

i) Testing Accuracy

It is used to determine the number of ECG signals that are identified precisely by the DRN, and is formulated as,

52 $$Acc = \frac{{t_{p} + t_{n} }}{{t_{p} + t_{n} + f_{p} + f_{n} }}$$

Here, $t_{p}$ specifies the total cases of ECG signals which are identified correctly with MI, $t_{n}$ is the count of the ECG signals categorized as normal, $f_{n}$ indicates the count of ECG records branded incorrectly as normal, and $f_{p}$ symbolizes the normal ECG signals that are identified as MI.

ii) Sensitivity

True Positive Rate (TPR) or sensitivity measures the count of ECG signals correctly identified with MI to the total number of ECG signals identified with MI, which is given by,

53 $$Sens = \frac{{t_{p} }}{{t_{p} + f_{n} }}$$

iii) Specificity

It is computed by determining the ratio of the ECG signals which is precisely identified as normal to the total number of ECG signals identified as normal. Specificity or True Negative Rate (TNR) is calculated based on the given expression

54 $$Spec = \frac{{t_{n} }}{{t_{n} + f_{p} }}$$

### Experimental results

**Fig. 3:**
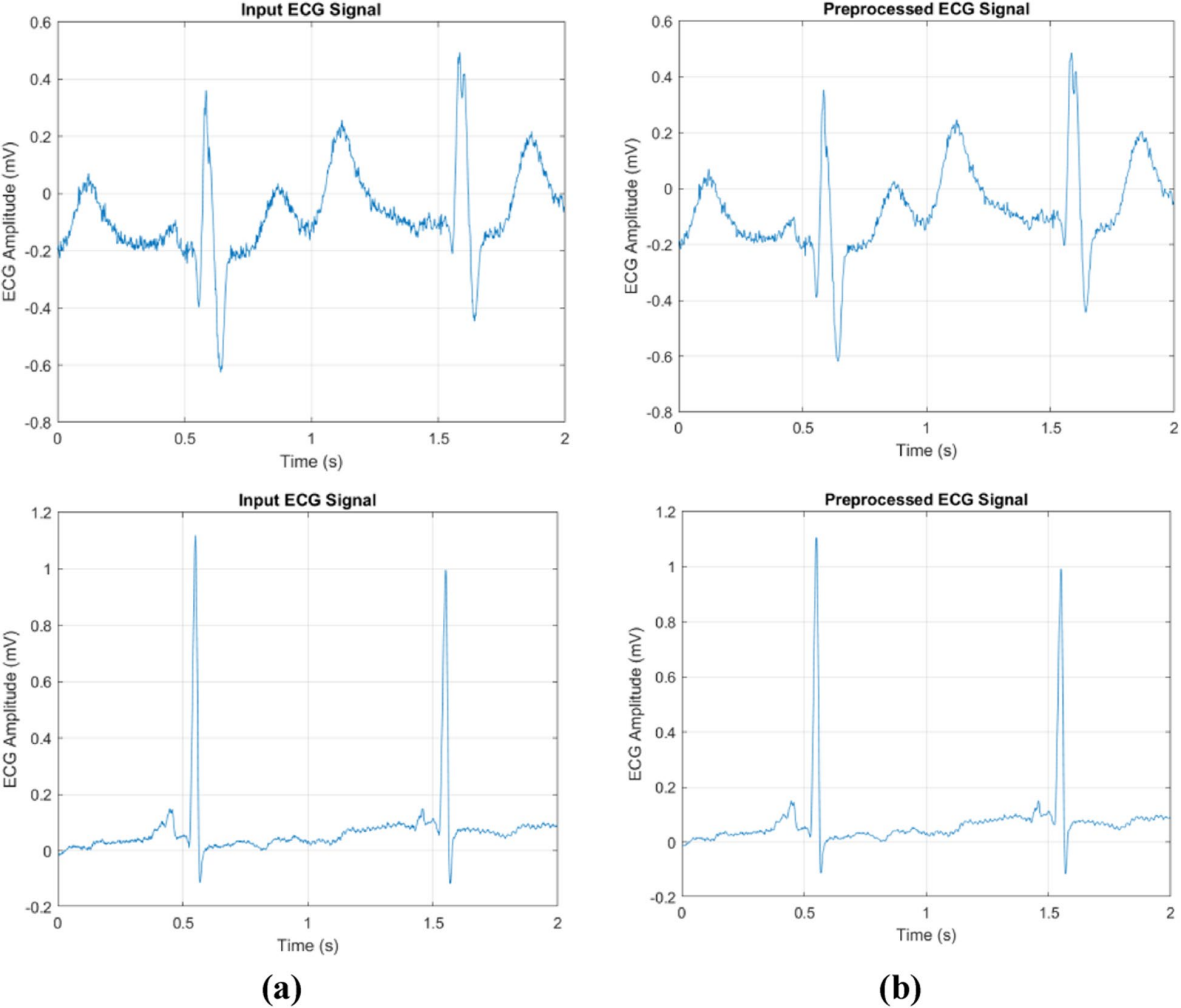
Experimental results **a**) Input **b**) Filtered signal using Median filter

Figure 3 shows the experimental results obtained by the SSS-DRN for MI detection. Here, Fig. 3 a) shows the input images, Fig. 3 b) denotes the filtering outputs obtained by the median filter.

## Performance evaluation

**Fig. 4:**
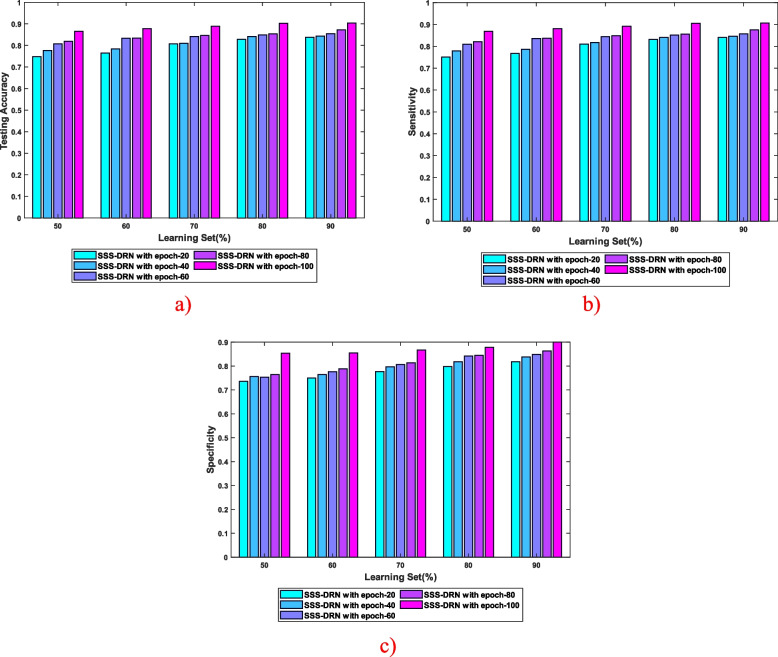
Performance assessment of the presented SSS-DRN considering **a**) testing accuracy, **b**) sensitivity, and **c**) specificity

Performance evaluation explicates the performance analysis of the formulated SSS-DRN for MI detection based on various parameters, considering different epochs, which is presented in Fig. 4. In Fig. 4 (a), the examination of the proposed SSS-DRN based on testing accuracy by altering the learning set is portrayed. The testing accuracy of the introduced SSS-DRN with a 70% learning set is 0.807, 0.810, 0.841, 0.846, and 0.888, corresponding to 20, 40, 60, 80, and 100 epochs. Figure 4 (b) displays the sensitivity-based evaluation of the developed SSS-DRN. At 80% learning set, the devised SSS-DRN computed a sensitivity of 0.831, 0.840, 0.852, 0.856, and 0.905, with 20, 40, 60, 80, and 100 epochs. The assessment of the presented SSS-DRN scheme for MI detection considering specificity is described in Fig. 4 (c). The value of specificity measured by the SSS-DRN with 90% learning set is 0.818 with 20 epochs, 0.838 with 40 epochs, and 0.849 with 60 epochs, 0.863 with 80 epochs, and 0.900 with 100 epochs.

### Algorithmic methods

In this work, the SSS algorithm is devised for the weight optimization of the DRN. The effectiveness of the SSS algorithm is investigated by comparing it with the existing algorithms, such as Particle Swarm Optimization (PSO) [20], Genetic Algorithm [29], Water Cycle Algorithm (WCA) [17], Smart Flower Optimization Algorithm (SFOA) [45], SSD [52], and SMO [7].

#### Algorithmic evaluation

**Fig. 5:**
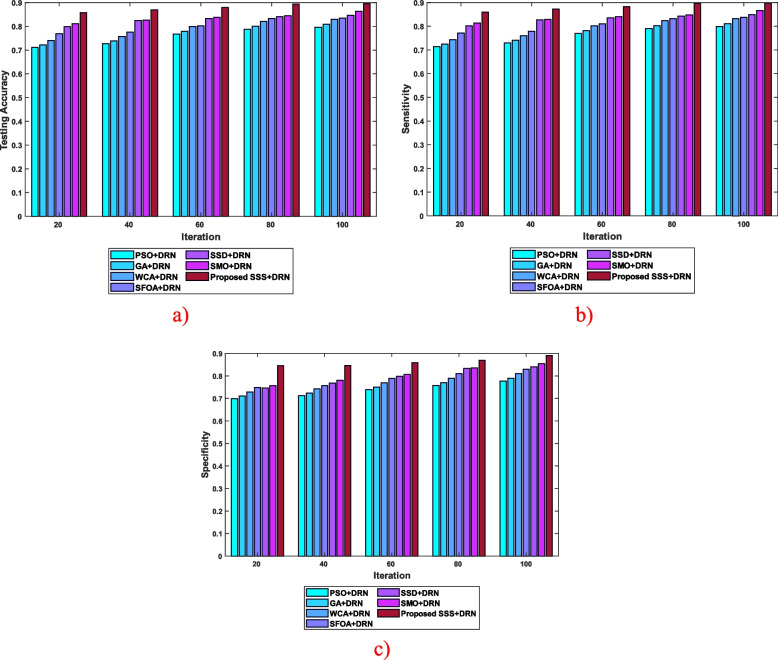
Algorithmic evaluation of the SSS algorithm based on dataset 1 **a**) testing accuracy, **b**) sensitivity, and **c**) specificity

The SSS algorithm developed in this work is correlated with the conventional algorithms here. Figure 5 presents the algorithmic assessment of the developed SSS algorithm by considering various parameters based on different iterations. In Fig. 5 (a), the formulated SSS algorithm is investigated considering the testing accuracy. With 40 iterations, the testing accuracy calculated by the various algorithms is 0.727 for PSO + DRN, 0.738 for GA + DRN, 0.757 for WCA + DRN, 0.776 for SFOA + DRN, 0.825 for SSD + DRN, 0.826 for SMO + DRN, and 0.869 for the devised SSS + DRN. Figure 5 (b) portrays the examination of the developed SSS algorithm concerning the sensitivity parameter. The value of sensitivity measured by the algorithms, like PSO + DRN, GA + DRN, WCA + DRN, SFOA + DRN, SSD + DRN, SMO + DRN, and the developed SSS + DRN is 0.770, 0.782, 0.802, 0.810, 0.835, 0.841, and 0.882, with 60 iterations. The investigation of the presented SSS algorithm based on the specificity is demonstrated in Fig. 5 (c). At 100 iterations, the different algorithms calculated specificity of 0.777 for PSO + DRN, 0.790 for GA + DRN, 0.810 for WCA + DRN, 0.830 for SFOA + DRN, 0.840 for SSD + DRN, 0.855 for SMO + DRN, and 0.891 for the developed SSS + DRN. These observations show that the presented SSS algorithm has achieved enhanced performance.

### Comparative techniques

The efficacy of the developed SSS-DRN is analysed with Minimum Skewness-Based Myocardial Infarction Detection Model (MSMIDM) [51], Support Vector Machine classification using the grasshopper optimization algorithm (SVM‑GOA) [44], CNN [1], kNN [36], Deep NN [30], ML-ResNet [25], and Hybrid approach of ResNet and Vision Transformer (ViT) models (hybrid ResNet-ViT model) [54].

### Comparative assessment

The comparative assessment of SSS-DRN based on different parameters, considering k-value and learning set for dataset 1 and dataset 2 are demonstrated as follows,

#### Evaluation with dataset 1

**Fig. 6:**
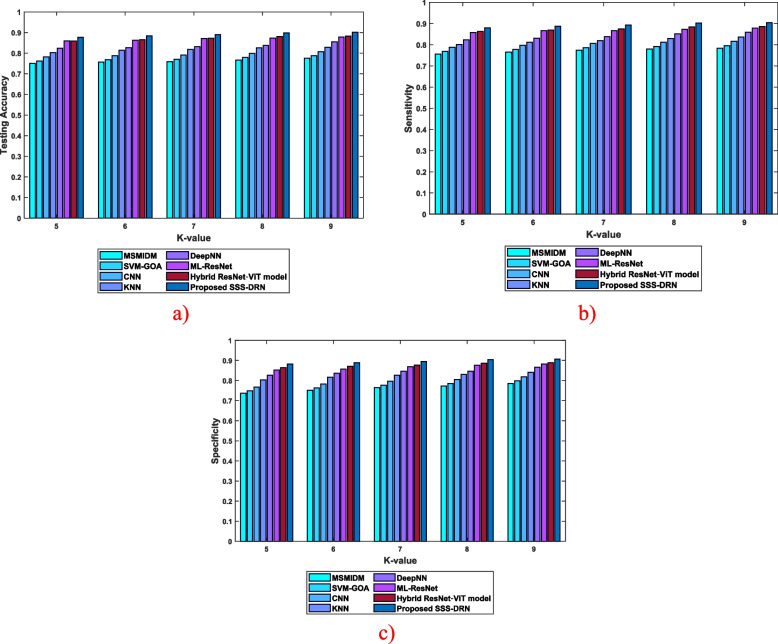
Comparative assessment of the proposed SSS-DRN technique concerning k-value with respect to **a**) accuracy, **b**) sensitivity, and **c**) specificity

**Fig. 7:**
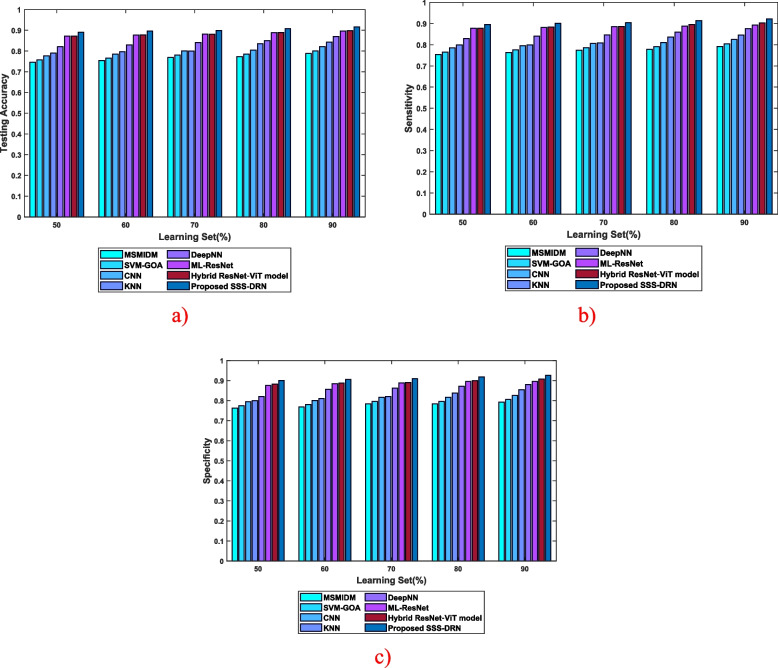
Comparative assessment of the devised SSS-DRN concerning learning set with respect to **a**) accuracy, **b**) sensitivity, and **c**) specificity

- Analysis considering k-value
  The efficiency of the SSS-DRN is evaluated based on k-value in this section by comparing it with the conventional schemes based on different parameters and it is demonstrated in Fig. 6. The comparative examination of the developed SSS-DRN based on testing accuracy is displayed in Fig. 6 (a). With k-value of 8, the prevailing schemes, like MSMIDM, SVM-GOA, CNN, kNN, Deep NN, ML-Res Net, and hybrid ResNet-ViT model achieved testing accuracies of 0.767, 0.779, 0.799, 0.826, 0.838, 0.873, and 0.881 correspondingly, while the presented SSS-DRN computed a high testing accuracy of 0.899. In Fig. 6 (b), the sensitivity-oriented investigation of the SSS-DRN is displayed. The sensitivity calculated by the proposed SSS-DRN is 0.893, while the traditional methods obtained a sensitivity of 0.774 for MSMIDM, 0.786 for SVM-GOA, 0.806 for CNN, 0.819 for kNN, 0.838 for Deep NN, 0.867 for ML-Res Net, and 0.875 for hybrid ResNet-ViT model, with k-value as 7. This shows that the developed SSS-DRN produced a variation of sensitivity value by 13.36%, 12.01%, 9.76%, 8.30%, 6.22%, 2.98%, and 2%. Figure 6 (c) illustrates the examination of the SSS-DRN with respect to specificity. The MI detection schemes, such as MSMIDM, SVM-GOA, CNN, kNN, Deep NN, ML-Res Net, hybrid ResNet-ViT model, and SSS-DRN measured specificity values of 0.751, 0.763, 0.783, 0.816, 0.836, 0.858, 0.871, and 0.889, for k-value of 6. This reveals that the devised SSS-DRN attained a high specificity with a variation of 15.46%, 14.14%, 11.93%, 8.20%, 5.94%, 3.52%, and 2% with the values attained by the available schemes.

b) Evaluation considering the learning set
  The assessment of the SSS-DRN model based on the learning set is given in Fig. 7. The analysis based on testing accuracy is depicted in Fig. 7 (a). The testing accuracy of the introduced SSS-DRN is 0.896, with a learning set of 60%, whereas the existing MI detection techniques, such as MSMIDM, SVM-GOA, CNN, kNN, Deep NN, ML-Res Net, and hybrid ResNet-ViT model attained testing accuracies of 0.754, 0.765, 0.785, 0.796, 0.830, 0.877, and 0.878, respectively. The devised scheme attained a variation of 15.85%, 14.53%, 12.34%, 11.10%, 7.36%, 2.12%, and 2% than the prevailing techniques. The assessment of the presented SSS-DRN concerning the sensitivity parameter is shown in Fig. 7 (b). The value of sensitivity calculated by the presented SSS-DRN is 0.904, with 70% learning set, which is higher than the sensitivity of 0.774, 0.786, 0.806, 0.809, 0.846, 0.885, and 0.886 computed by the schemes such as MSMIDM, SVM-GOA, CNN, kNN, Deep NN, ML-Res Net, and hybrid ResNet-ViT model by 14.45%, 13.12%, 10.89%, 10.54%, 6.40%, 2.09%, and 2%, respectively. The specificity-based assessment of the developed SSS-DRN MI detection scheme is displayed in Fig. 7. With 80% learning set, the different MI identification techniques, like MSMIDM, SVM-GOA, CNN, kNN, Deep NN, ML-Res Net, hybrid ResNet-ViT model, and developed SSS-DRN computed a specificity of 0.784, 0.796, 0.816, 0.838, 0.871, 0.895, 0.9, and 0.918. This reveals that the presented SSS-DRN produced an enhancement in performance by 14.64%, 13.31%, 11.09%, 8.72%, 5.10%, 2.47%, and 2%.

### Evaluation with dataset 2

**Fig. 8:**
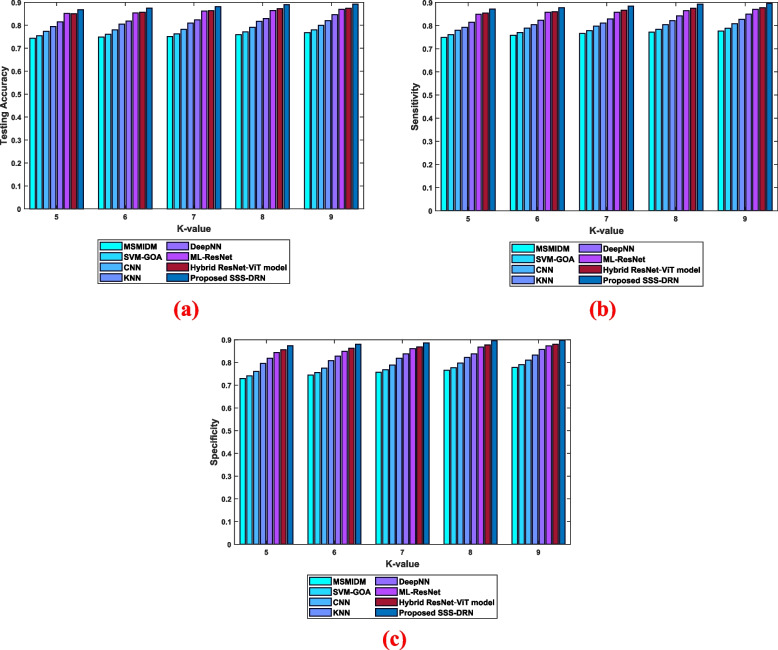
Comparative analysis of the devised SSS-DRN based on k value concerning dataset 2 **a**) accuracy, **b**) sensitivity, and **c**) specificity

**Fig. 9:**
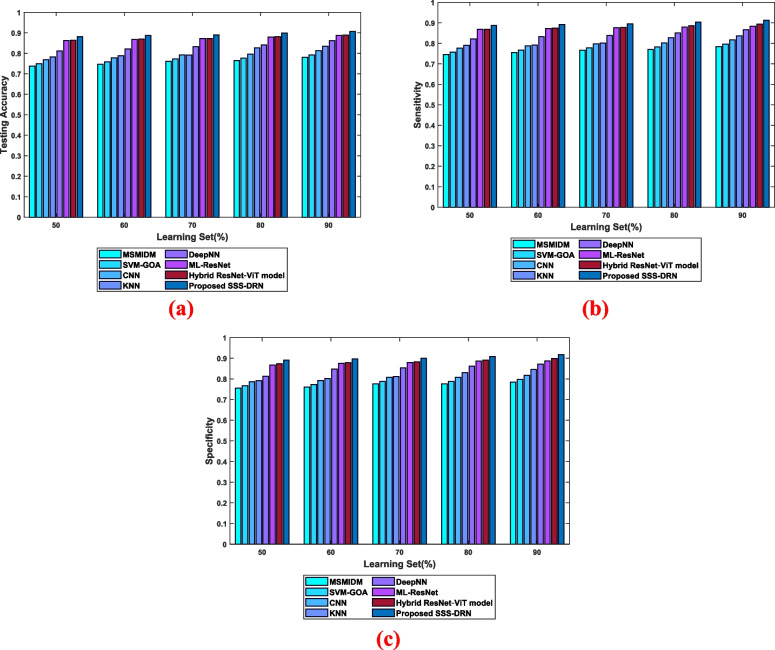
Comparative analysis of the devised SSS-DRN based on learning set concerning dataset 2 **a**) accuracy, **b**) sensitivity, and **c**) specificity

- assessment regarding K-value
  Figure 8 represents the evaluation of the SSS-DRN model based on dataset 2. The analysis regarding testing accuracy is depicted in Fig. 8 a). When considering the k value as 9, the devised SSN-DRN model obtained an accuracy of value 0.892. The existing models like MSMIDM, SVM-GOA, CNN, kNN, Deep NN, ML-Res Net, and hybrid ResNet-ViT models are 0.768, 0.780, 0.800, 0.820, 0.846, 0.870, and 0.874. The sensitivity evaluation of the proposed SSN-DRN model is depicted in Fig. 8 b). With k value 9, the sensitivity values noted by the MSMIDM, SVM-GOA, CNN, kNN, Deep NN, ML-Res Net, hybrid ResNet-ViT model, and devised SSN-DRN are 0.776, 0.788, 0.808, 0.827, 0.850, 0.870, 0.878, and 0.896, respectively. Figure 8 c) depicts the assessment regarding specificity. The specificity value of the devised SSN-DRN is 0.898, while the traditional models like MSMIDM, SVM-GOA, CNN, kNN, Deep NN, ML-Res Net, and hybrid ResNet-ViT model noted specificity values of 0.778, 0.790, 0.810, 0.832, 0.857, 0.873, and 0.880, respectively.

b) Evaluation based on the learning set
  The efficiency of the SSS-DRN is evaluated based on the learning set and the comparison of the devised model with conventional schemes is shown in Fig. 9. The comparative examination of the developed SSS-DRN based on testing accuracy is displayed in Fig. 9(a). when 90% of training data the prevailing schemes, like MSMIDM, SVM-GOA, CNN, kNN, Deep NN, ML-Res Net, and hybrid ResNet-ViT model achieved testing accuracies of 0.780, 0.792, 0.813, 0.835, 0.861, 0.887, and 0.889 correspondingly, while the presented SSS-DRN computed a high testing accuracy of 0.907. In Fig. 9 (b), the sensitivity of the SSS-DRN is displayed. The sensitivity calculated by the proposed SSS-DRN is 0.912, while the traditional methods obtained a sensitivity of 0.784 for MSMIDM, 0.796 for SVM-GOA, 0.817 for CNN, 0.837 for kNN, 0.867 for Deep NN, 0.884 for ML-Res Net, and 0.894 for hybrid ResNet-ViT model when considering 90% training data. Figure 9 (c) illustrates the examination of the SSS-DRN with respect to specificity. The MI detection schemes, such as MSMIDM, SVM-GOA, CNN, kNN, Deep NN, ML-Res Net, hybrid ResNet-ViT model, and SSS-DRN measured specificity with a range 0.785, 0.797, 0.818, 0.846, 0.871, 0.887, 0.898, and 0.917 for 90% training data.

### Comparative discussion

The efficacy of the devised SSS-DRN is investigated in this section by comparing it with the conventional MI identification methods, like MSMIDM, SVM-GOA, CNN, kNN, Deep NN, ML-Res Net, and hybrid ResNet-ViT model and this is displayed in Table 1. The analysis is accomplished on the basis of various evaluation measures considering various values of k-value and learning set. Table 1 reveals the values of the three performance measures utilized in the analysis corresponding to a k-value of 9 and a learning set of 90%. The developed SSS-DRN obtained testing accuracy of 0.916, due to the utilization of numerous discriminative features during the identification process. Further, a higher sensitivity value of 0.921 is found to have been measured by the devised SSS-DRN owing to the use of DRN for classification. Moreover, the weight optimization of DRN based on the proposed SSS algorithm has contributed to a maximal specificity value of 0.926.

**Table 1:** Comparative discussion

*Datasets*	*Variations*	*Metrics*	MSMIDM	SVM-GOA	*CNN*	*kNN*	*Deep NN*	*ML-ResNet*	hybrid ResNet-ViT model	*Proposed SSS-DRN*
***Dataset 1***	***k-value***	***Testing Accuracy***	0.775	0.787	0.808	0.829	0.854	0.878	0.883	0.901
		***Sensitivity***	0.784	0.796	0.816	0.836	0.859	0.879	0.887	0.905
		***Specificity***	0.786	0.798	0.819	0.841	0.866	0.882	0.889	0.907
	***Learning set***	***Testing Accuracy***	0.788	0.800	0.821	0.843	0.870	0.896	0.898	**0.916**
		***Sensitivity***	0.792	0.804	0.825	0.845	0.875	0.893	0.903	**0.921**
		***Specificity***	0.793	0.805	0.826	0.854	0.880	0.896	0.908	**0.926**
***Dataset 2***	***k-value***	***Testing Accuracy***	0.768	0.780	0.800	0.820	0.846	0.870	0.874	0.892
		***Sensitivity***	0.776	0.788	0.808	0.827	0.850	0.870	0.878	0.896
		***Specificity***	0.778	0.790	0.810	0.832	0.857	0.873	0.880	0.898
	***Learning set***	***Testing Accuracy***	0.780	0.792	0.813	0.835	0.861	0.887	0.889	0.907
		***Sensitivity***	0.784	0.796	0.817	0.837	0.867	0.884	0.894	0.912
		***Specificity***	0.785	0.797	0.818	0.846	0.871	0.887	0.898	0.917

### Ablation study

**Fig. 10:**
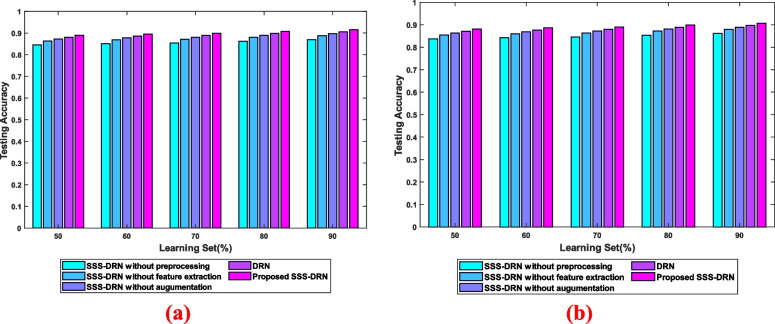
Ablation assessment of the devised SSS-DRN model based on accuracy **a**) Dataset 1 and **b**) Dataset 2

The ablation study refers to the removal of certain components from the model in order to analyse the impact on the model’s performance to understand the contribution of removed component. Figure 10 elucidates the ablation assessment of the devised SSS-DRN model concerning accuracy. The evaluation with respect to dataset 1 is interpreted in Fig. 10 a). With 90% training data, the proposed SSS-DRN model obtained testing accuracy values of 0.916 and the accuracy values noted by the SSS-DRN without pre-processing, SSS-DRN without feature extraction, SSS-DRN without image augmentation, and DRN are 0.870, 0.888, 0.898, and 0.9067, respectively. Figure 10 b) represents the ablation study of the devised model for dataset 2. The testing accuracy reached by the SSS-DRN without pre-processing, SSS-DRN without feature extraction, SSS-DRN without image augmentation, DRN, and SSS-DRN model are 0.861, 0.879, 0.889, 0.897, and 0.907, respectively concerning the training data as 90%.

### Analysis by varying noise

**Fig. 11:**
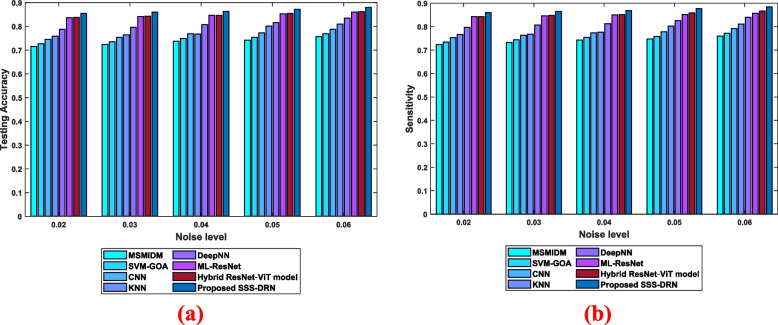
Analysis of the devised SSS-DRN by varying noise levels **a**) Dataset 1, **b**) Dataset 2

Figure 11 depicts the assessment by considering the impact of noise. The analysis regarding dataset 1 is provided in Fig. 11 a). By considering the noise level as 0.06, the conventional schemes and the devised SSS-DRN model obtained testing accuracy of 0.756, 0.768, 0.788, 0.809, 0.835, 0.860, 0.862, and 0.879. This shows that the performance gain observed by the devised model is 13.96%, 12.61%, 10.37%, 7.95%, 5%, 2.21%, and 2% improved than the traditional models like MSMIDM, SVM-GOA, CNN, kNN, Deep NN, ML-Res Net, and hybrid ResNet-ViT model. The analysis of the devised moel by varying noise levels for dataset 2 is interpreted in Fig. 11 b). The testing accuracy reached by the MSMIDM, SVM-GOA, CNN, kNN, Deep NN, ML-Res Net, and hybrid ResNet-ViT models is 0.760, 0.772, 0.792, 0.811, 0.840, 0.857, and 0.867 whereas the devised model obtained testing accuracy values of 0.885 for the noise level 0.06.

### Assessment by varying features

**Fig. 12:**
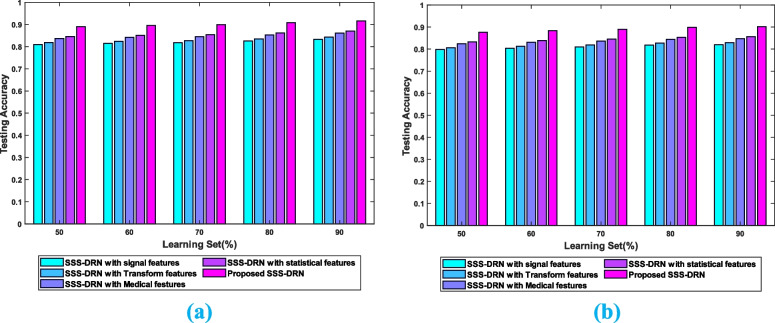
Analysis of the devised SSS-DRN by varying features **a**) Dataset 1, **b**) Dataset 2

The analysis by considering different features of the SSS-DRN model is depicted in Fig. 12. The impact of features for dataset 1 of the devised scheme is elucidated in Fig. 12 a). with 90% of data, the SSS-DRN withs signal features, SSS-DRN with transform features, SSS-DRN with medical features, SSS-DRN with statistical features, and the devised model SSS-DRN (with all features) obtained testing accuracy of 0.833, 0.843, 0.861, 0.870, and 0.916. Figure 12 b) depicts the assessment by varying different features for dataset 2. The SSS-DRN with signal features, SSS-DRN with transform features, SSS-DRN with medical features, SSS-DRN with statistical features, and the devised model SSS-DRN (with all features) reached testing accuracy of 0.820, 0.829, 0.847, 0.856, and 0.901 for the training data 90%.

### Confusion matrix

**Fig. 13:**
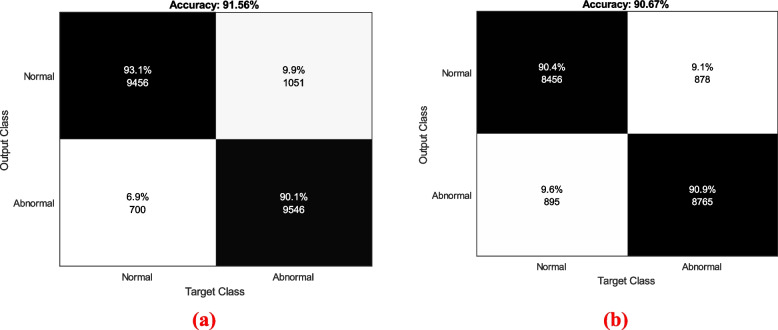
Confusion matrix of the devised SSS-DRN **a**) Dataset 1 **b**) Dataset 2

A confusion matrix represents a table that is used for accessing the performance of the classification model based on the comparison of predicted and true labels. Here, the actual classes are provided in rows and the predicted classes are represented in columns. It provides the counts of True positives (TP), true negatives (TN), False positives (FP), and false negatives (FN). Here, the cell is filled by the count of data points that belong to the combination of actual and predicted classes. Here, the correct predictive positive cases by the model are indicated as TP, the correct predicted negative cases are represented in TN, FP indicates the incorrectly predicted positive cases, and the incorrectly predicted negative cases are signified as FN. Figure 13 represents the confusion matrix of dataset 1 and dataset 2. Figure 13 a) shows the confusion matrix of dataset 1. The accuracy value noted by Dataset 1 is 91.56%. Here, the total samples in TP are 9456, TN is 9546, FP is 1051, and FN is 700. The confusion matrix of dataset 2 is provided in Fig. 13 b). Here, the accuracy value is 90.67%. Also, the samples in TP are 8456, TN is 8765, FP is 878, and FN is 895.

#### Convergence graph

**Fig. 14:**
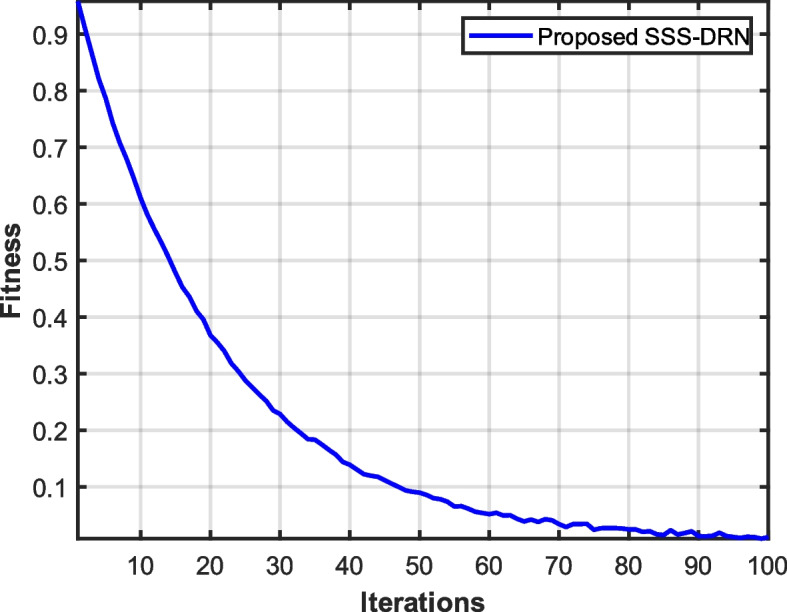
Convergence analysis of the devised SSS-DRN model

The convergence graph helps to analyse how well the devised algorithm performs over the changing iterations. It identifies whether the obtained solution is a feasible good solution or it stuck to local optima thereby providing the details about the solution whether it is stable or not. Figure 14 represents the convergence graph of the devised SSS-DRN. When the iteration is 100, the fitness obtained by the devised model is 0.011. The faster convergence with low fitness value signifies that the devised algorithm is effective in obtaining optimal solutions.

### Receiver operating characteristic (ROC) curve analysis

**Fig. 15:**
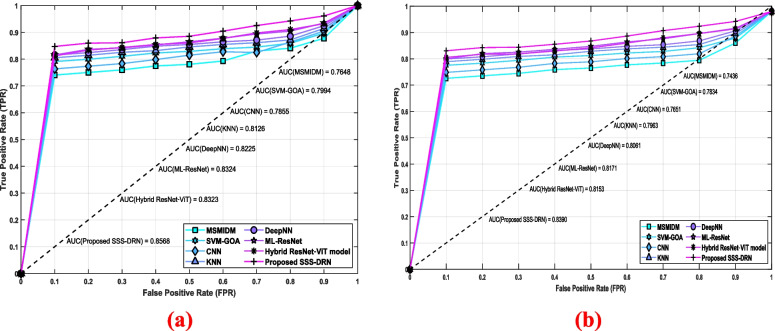
ROC Curve Analysis of the devised SSS-DRN model **a**) Dataset 1 **b**) Dataset 2

The ROC curve is the graphical model that is used for analyzing the performance of the binary classifier of the model at different threshold values. The ROC curve is provided by calculating the TPR and FPR are each threshold setting. It indicates the trade-off between the sensitivity and specificity of the classifier. Figure 15 shows the ROC curve analysis of the devised scheme. Figure 15 a) shows the ROC curve assessment for dataset 1. When the FPR is considered as 0.5, the TPR values noted by the MSMIDM, SVM-GOA, CNN, kNN, Deep NN, ML-Res Net, hybrid ResNet-ViT model and the devised SSS-DRN model are 0.781, 0.829, 0.815, 0.846, 0.855, 0.865, 0.861, and 0.885. Figure 15 b) shows the ROC curve evaluation based on dataset 2. With FPR value 0.5, the TPR reached by the MSMIDM, SVM-GOA, CNN, kNN, Deep NN, ML-Res Net, hybrid ResNet-ViT model, and SSS-DRN are 0.765, 0.813, 0.789, 0.829, 0.838, 0.847, 0.842, and 0.868, respectively. The high TPR values noted by the devised model that the correctly identify the positive cases.

### Computational complexity evaluation

The computational complexity refers to the total time required by the devised model to complete a particular time. Table 2 shows the computational time of the devised SSS-DRN model. The devised method obtained a minimum time of 7.0959 s for dataset 1 and 8.0457 s for dataset 2. From the analysis, the devised model provides quick decisions as the total time obtained by the devised model is minimal compared to other models. This signifies that the model is highly scalable and this makes the model handle larger datasets.

**Table 2:** Computational Complexity

Method	Time (sec)	
	**Dataset 1**	**Dataset 2**
MSMIDM	14.5968	15.4869
SVM-GOA	13.0957	14.4699
CNN	12.5097	13.9569
KNN	11.9865	12.0095
Deep NN	9.0947	10.0095
ML-Res Net	8.0959	9.4596
Hybrid ResNet-ViT model	8.0123	9.1245
Proposed SSS-DRN	**7.0959**	**8.0457**

### Analysis of variance (ANOVA) analysis

ANOVA test is a statistical test employed to assess the difference between the mean of more than two groups. The ANOVA effectively handles multiple factors and their connections thereby providing a robust way for understanding the intricate relationship. Table 3 shows the ANOVA analysis of the devised scheme. Here, the first column represents the independent variables along with the model error. The Df column signifies the degree of freedom for the independent variable and the residuals. The sum of squares indicates the total variation among the group means and the overall mean represented by the variable. The high value of F represents that there is a larger difference between the group of means compared to the variations within the group. The p-value noted by the devised model is less than 0.05, which represents that the devised model rejects the null hypothesis and signifies that there is a significant difference among the group means.

**Table 3:** ANOVA Analysis

	**Sum of squares**	**Degrees of freedom (Df)**	**F**	**P-value**
C	0.145764	4	17.56752	0.000258
Residual	0.213424	120		

### SHAP (SHapley Additive exPlanations) visualization

**Fig. 16:**
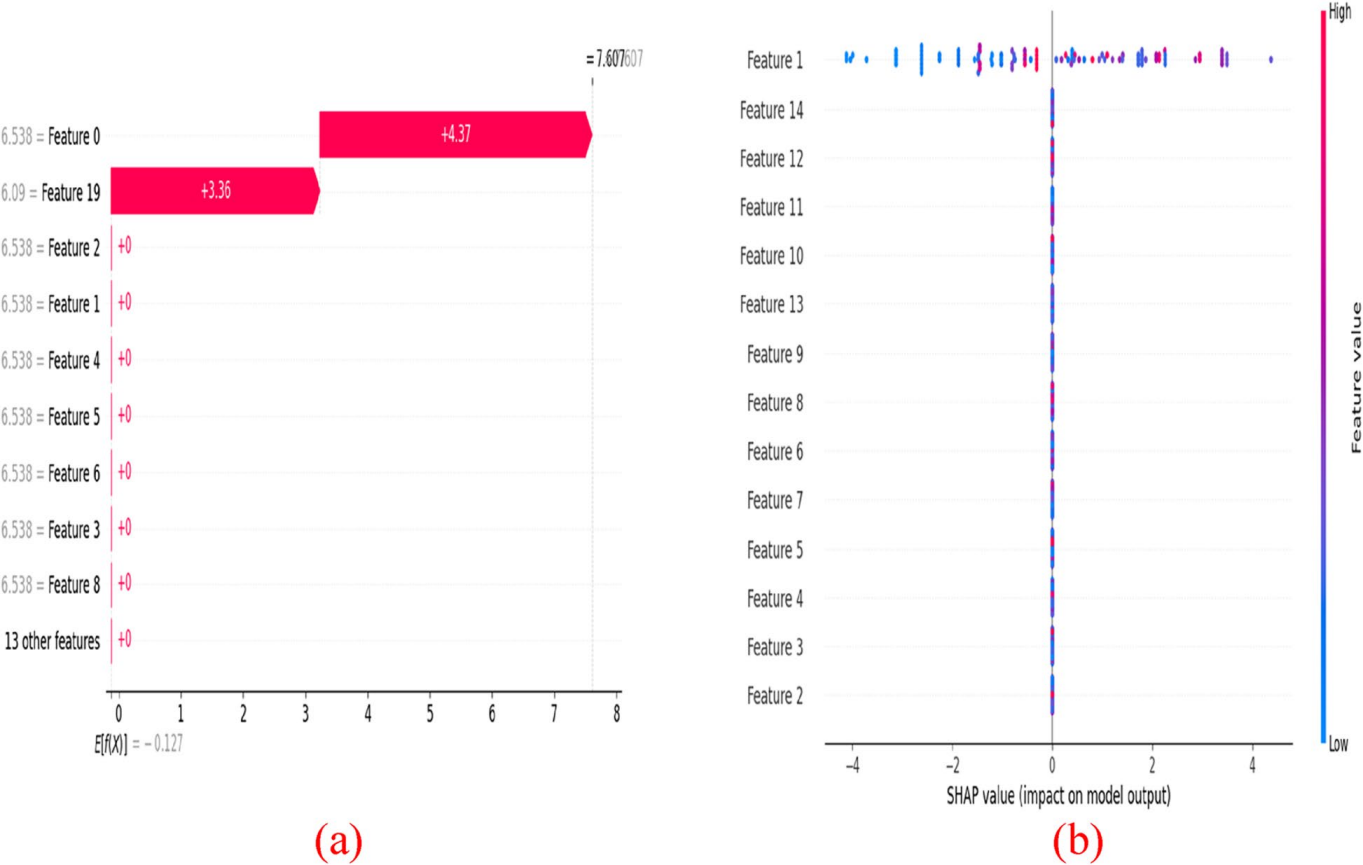
SHAP visualization of the devised SSS-DRN model **a**) Waterfall plot **b**) summary plot

The SHAP model is the visual way of explaining the output of the devised model. It uses a game-theoretic approach and measures how each feature contributes to the final output. In DL schemes, SHAP values show how each feature affects each final prediction, the significance of each feature compared to others, and the model's reliance on the interaction between features. The SHAP measures the importance of each feature in the model’s prediction based on Shapley values. Figure 16 provides the SHAP visualization of the devised SSS-DRN model. Figure 16 a) shows the waterfall plot of the devised scheme. Here, the contribution of each feature in specific prediction is illustrated in this waterfall plot. Here, the starting point specifies the base value and the red bars indicate positive contributions, which increases the predicted value. Figure 16 b) represents the summary plot of the devised scheme. Here, the feature names with their importance from top to bottom are represented in the Y-axis and the SHAP values are indicated in the X-axis. The row of data obtained from the original dataset is represented in point.

## Conclusion

In this research, the SSS-DRN model is implemented for MI detection from ECG. MI is considered as a life-threatening disease that can significantly cause total death or damage to the heart and hence requires to be identified as early as possible. Here, a novel DL-based technique is devised using DRN, whose weight parameters are adapted using the developed SSS algorithm. The ECG signals are initially subjected to a median filter, followed by feature extraction, wherein multiple discriminative features are determined. The feature vector produced is then forwarded to the data augmentation phase, where the feature is increased by permutation, random generation, and re-sampling. Finally, MI identification is accomplished utilizing the devised SSS-DRN. Here, the SSS algorithm is formulated based on the SSD and SMO algorithms. Moreover, the developed SSS-DRN is investigated for efficiency considering metrics, like accuracy, sensitivity, and specificity and obtained values of 0.916, 0.921, and 0.926. The devised model could be embedded in real-time clinical settings like hospital ECG machines, portable wearable ECG monitors, and mobile health applications. The devised scheme highly prevents overfitting and computational issues, which makes it highly suitable for real-time clinical systems. This improves the clinical decision-making process with increased patient outcomes. The devised model cannot be deployed in real-time applications and the devised model takes a long time to differentiate distinct heartbeat from ECG that contains noise. Although the devised model performs well still the computational cost of the DRN remains a major challenge of the model. Further research directions include the utilization of advanced features to augment the efficiency of the approach and the application of the technique to identify other heart diseases like arrhythmias, atrial fibrillation, and heart failure. Also, we will further explore about the integration of ECG with echocardiography, patient history, or genetic biomarkers for improved diagnostics. Additionally, the devised model will be embedded in real-time monitoring systems for analysing at-risk patients thereby improving the early detection of cardiac diseases and preventing further complications. In future, we plan to integrate the transfer learning approaches for tuning the model and will further test the model on different datasets. This integration with the devised model will make the model adaptable to identify individual patient variations.

## Data Availability

No datasets were generated or analysed during the current study. The data that support the findings of this study are openly available in PTB Diagnostic ECG Database at https://www.physionet.org/content/ptbdb/1.0.0/, reference number [42] and MIT-BIH Arrhythmia Database at https://www.kaggle.com/datasets/taejoongyoon/mitbit-arrhythmia-database, reference number [53].

## References

[1] Alghamdi A, Hammad M, Ugail H, Abdel-Raheem A, Muhammad K, Khalifa HS, Abd El-Latif AA. Detection of myocardial infarction based on novel deep transfer learning methods for urban healthcare in smart cities. Multimedia tools and applications. 2020;83(5):14913-34.

[2] Alkhammash EH, Assiri SA, Nemenqani DM, Althaqafi RMM, Hadjouni M, Saeed F, Elshewey AM. Application of Machine Learning to Predict COVID-19 Spread via an Optimized BPSO Model. Biomimetics. 2023;8(6):457. 37887588 10.3390/biomimetics8060457 PMC10604133

[3] Alkhammash EH, Kamel AF, Al-Fattah AM, Elshewey AM. Optimized Multivariate Adaptive Regression Splines for Predicting Crude Oil Demand in Saudi Arabia. Discret Dyn Nat Soc. 2022;12:1–9.

[4] Alzakari SA, Alhussan AA, Qenawy AT, Elshewey AM. Early Detection of Potato Disease Using an Enhanced Convolutional Neural Network-Long Short-Term Memory Deep Learning Model. Potato Research. 2024.

[5] Arif M, Malagore IA, Afsar FA. Automatic detection and localization of myocardial infarction using back propagation neural networks. In proceedings of 2010 4th International Conference on Bioinformatics and Biomedical Engineering. 2010;1–4.

[6] Arif M, Malagore IA, Afsar FA. Detection and localization of myocardial infarction using k-nearest neighbor classifier. J Med Syst. 2012;36(1):279–89. 20703720 10.1007/s10916-010-9474-3

[7] Bansal JC, Sharma H, Jadon SS, Clerc M. Spider monkey optimization algorithm for numerical optimization. Memetic computing. 2014;6(1):31–47.

[8] Bellfield RA, Ortega-Martorell S, Lip GY, Oxborough D, Olier I. Impact of ECG data format on the performance of machine learning models for the prediction of myocardial infarction. J Electrocardiol. 2024;84:17–26. 38471239 10.1016/j.jelectrocard.2024.03.005

[9] Bender T, Beinecke JM, Krefting D, Muller C, Dathe H, Seidler T, Spicher N, Hauschild AC. Analysis of a Deep Learning Model for 12- lead ECG Classification Reveals Learned Features similar to Diagnostic Criteria. IEEE Journal of Biomedical and Health Infomatics. 2023;28(4):1848–59. 10.1109/JBHI.2023.3271858 37126621

[10] Bhagyalakshmi V, Pujeri RV, Devanagavi GD. GB-SVNN: Genetic BAT assisted support vector neural network for arrhythmia classification using ECG signals. Journal of King Saud University-Computer and Information Sciences. 2021;33(1):54–67.

[11] Chen Z, Chen Y, Wu L, Cheng S, Lin P. Deep residual network based fault detection and diagnosis of photovoltaic arrays using current-voltage curves and ambient conditions. Energy Convers Manage. 2019;198: 111793.

[12] Dar MN, Akram MU, Shaukat A, Khan MA. ECG based biometric identification for population with normal and cardiac anomalies using hybrid HRV and DWT features. In proceedings of 2015 5th International Conference on IT Convergence and Security (ICITCS). 2015;1–5.

[13] Deepika S, Jaisankar N. 2024. Detecting and classifying Myocardial Infarction in Echocardiogram Frames with an Enhanced CNN Algorithm and ECV-3D Network. IEEE Access.

[14] Djaafari A, Ibrahim A, Bailek N, Bouchouicha K, Hassan MA, Kuriqi A, Al-Ansari N, El-kenawy EM. Hourly predictions of direct normal irradiation using an innovative hybrid LSTM model for concentrating solar power projects in hyper-arid regions. Energy Rep. 2022;8:15548–62.

[15] Elshewey AM, Shams MY, Tawfeek SM, Alharbi AH, Ibrahim A, Abdelhamid AA, Eid MM, Khodadadi N, Abualigah L, Khafaga DS, Tarek Z. Optimizing HCV Disease Prediction in Egypt: The hyOPTGB Framework. Diagnostics. 2023;13(22):3439. 37998575 10.3390/diagnostics13223439 PMC10670002

[16] Elshewey AM, Tawfeek SM, Alhussan AA, Radwan M, Abed AH. 2024. Optimized Deep Learning for Potato Blight Detection Using the Waterwheel Plant Algorithm and Sine Cosine Algorithm. Potato Research. 2024:1-25.

[17] Eskandar H, Sadollah A, Bahreininejad A, Hamdi M. Water cycle algorithm–A novel metaheuristic optimization method for solving constrained engineering optimization problems. Comput Struct. 2012;110:151–66.

[18] Faragallah OS. Robust noise MKMFCC–SVM automatic speaker identification. Int J Speech Technol. 2018;21(2):185–92.

[19] Gaber KS, Singla MK. Predictive Analysis of Groundwater Resources Using Random Forest Regression. Journal of Artificial Intelligence and Metaheuristics. 2025;9(1):11–9.

[20] Gad AG. Particle Swarm Optimization Algorithm and Its Applications: A Systematic Review. Archives of Computational Methods in Engineering. 2022;29:2531–61.

[21] Gohel B, Tiwary US, Lahiri T. Relative amplitude based features of characteristic ECG-peaks for identification of coronary artery disease. In Proceedings of the First International Conference on Intelligent Human Computer Interaction, Springer, New Delhi. 2009;140–146.

[22] Golande AL, Pavankumar T. Optical Electrocardiogram Based Heart Disease Prediction using Hybrid Deep Learning. Journal of Bigdata. 2023;10(1):189.

[23] Guo Y, Du GQ, Shen WQ, Du C, He PN, Siuly S. Automatic myocardial infarction detection in contrast echocardiography based on polar residual network. Comput Methods Programs Biomed. 2021;198: 105791. 33080493 10.1016/j.cmpb.2020.105791

[24] Hammad M, Alkinani MH, Gupta BB, El-Latif A, Ahmed A. Myocardial infarction detection based on deep neural network on imbalanced data. Multimedia Syst. 2022;28(4):1373–85.

[25] Han C, Shi L. ML–ResNet: A novel network to detect and locate myocardial infarction using 12 leads ECG. Comput Methods Programs Biomed. 2020;185: 105138. 31669959 10.1016/j.cmpb.2019.105138

[26] Hao P, Gao X, Li Z, Zhang J, Wu F, Bai C. Multi-branch fusion network for Myocardial infarction screening from 12-lead ECG images. Comput Methods Programs Biomed. 2020;184: 105286. 31891901 10.1016/j.cmpb.2019.105286

[27] Hemanth DJ. EEG signal based modified Kohonen neural networks for classification of human mental emotions. Journal of Artificial Intelligence and Systems. 2020;2:1–13.

[28] Huang KC, Lin DSH, Jeng GS, Lin TT, Lin LY, Lee CK, Lin LC. Left Ventricular Segmentation, Warping, and Myocardial Registration for Automated Strain Measurement. J Imaging Inform Med. 2024;37(5):2274-86. 10.1007/s10278-024-01119-5 PMC11522271 38639806

[29] Immanuel SD, Chakraborty UK. Genetic Algorithm: An Approach on Optimization. Coimbatore: International Conference on Communication and Electronics Systems (ICCES), IEEE; 2019. p. 701–708.

[30] Jafarian K, Vahdat V, Salehi S, Mobin M. Automating detection and localization of myocardial infarction using shallow and end-to-end deep neural networks. Appl Soft Comput. 2020;93: 106383.

[31] Janse MJ, Capucci A, Coronel R, Fabius MAW. Variability of recovery of excitability in the normal canine and the ischaemic porcine heart. Eur Heart J. 1985;6:41–52. 2417853 10.1093/eurheartj/6.suppl_d.41

[32] Bax JJ, Baumgartner H, Ceconi C, Dean V, Deaton C, Fagard R, Funck-Brentano C, Hasdai D, Hoes A, Kirchhof P. Third universal definition of myocardial infarction. J Am Coll Cardiol. 2012;60(16):1581–98. 22958960 10.1016/j.jacc.2012.08.001

[33] Khafaga DS, Alhussan AA, El-kenawy EM, Takieldeen AE, Hassan TM, Hegazy EA, Eid EAF, Ibrahim A, Abdelhamid AA. Meta-heuristics for Feature Selection and Classification in Diagnostic Breast Cancer. Computers, Materials & Continua. 2022;73(1):749–65.

[34] Khafaga DS, Alhussan AA, El-Kenawy ESM, Ibrahim A, Eid MM, Abdelhamid AA. Solving optimization problems of metamaterial and double T-shape antennas using advanced meta-heuristics algorithms. IEEE Access. 2022;10:74449–71.

[35] Lin WH, Wong MYM, Pu LN, Zhang YT. 2010. Comparison of median filter and discrete dyadic wavelet transform for noise cancellation in electrocardiogram. In Proceedings of 2010 Annual International Conference of the IEEE Engineering in Medicine and Biology, IEEE; 2010. p. 2395–2398. 10.1109/IEMBS.2010.5627195 21096585

[36] Lin Z, Gao Y, Chen Y, Ge Q, Mahara G, Zhang J. Automated detection of myocardial infarction using robust features extracted from 12-lead ECG. Signal, Image and Video Processing. 2020;14:857-65.

[37] Liu J, Zhang C, Zhu Y, Ristaniemi T, Parviainen T, Cong F. Automated detection and localization system of myocardial infarction in single-beat ECG using Dual-Q TQWT and wavelet packet tensor decomposition. Comput Methods Programs Biomed. 2020;184: 105120. 31627147 10.1016/j.cmpb.2019.105120

[38] Manikandan MS, Dandapat S. Wavelet energy based diagnostic distortion measure for ECG. Biomed Signal Process Control. 2007;2(2):80–96.

[39] Mohamed ME. A Review on Waste Management Techniques for Sustainable Energy Production. Metaheuristic Optimization Review. 2025;3(2):47–58.

[40] Nayak BP, Kar S, Routray A, Padhi AK. A biomedical approach to retrieve information on driver's fatigue by integrating EEG, ECG and blood biomarkers during simulated driving session. In Proceedings of 2012 4th International Conference on Intelligent Human Computer Interaction (IHCI), IEEE; 2012. p. 1–6.

[41] Pan Q, Li X, Fang L. Data Augmentation for Deep Learning-Based ECG Analysis. In proceedings of Feature Engineering and Computational Intelligence in ECG Monitoring. 2020;91–111.

[42] PTB Diagnostic ECG Database is gathered from https://www.physionet.org/content/ptbdb/1.0.0/ accessed on March 2021.

[43] Safdar MF, Pałka P, Nowak RM, Al FA. A novel data augmentation approach for enhancement of ECG signal classification. Biomed Signal Process Control. 2023;86:105114.

[44] Safdarian N, Nezhad SYD, Dabanloo NJ. Detection and Classification of Myocardial Infarction with Support Vector Machine Classifier Using Grasshopper Optimization Algorithm. Journal of Medical Signals & Sensors. 2021;11(3):185–93. 34466398 10.4103/jmss.JMSS_24_20 PMC8382032

[45] Sattar D, Salim R. A smart metaheuristic algorithm for solving engineering problems. Engineering with Computers. 2021;37(3):2389–417.

[46] Sharma LD, Sunkaria RK. Myocardial infarction detection and localization using optimal features based lead specific approach. IRBM. 2020;41(1):58–70.

[47] Sharma M, San Tan R, Acharya UR. A novel automated diagnostic system for classification of myocardial infarction ECG signals using an optimal biorthogonal filter bank. Comput Biol Med. 2018;102:341–56. 30049414 10.1016/j.compbiomed.2018.07.005

[48] Sun Q, Wang Q, Ji B, Wu W, Huang W, Wang C. The Cardiodynamicsgram Based Early Detection of Myocardial Ischemia Using the Lempel-Ziv Complexity. IEEE Access. 2020;8:207894–904.

[49] Swain SS, Patra D, Singh YO. Automated detection of myocardial infarction in ECG using modified Stockwell transform and phase distribution pattern from time-frequency analysis. Biocybernetics and Biomedical Engineering. 2020;40(3):1174–89.

[50] Swenne CA, Ter Haar CC. 2023. Context-independent identification of myocardial ischemia in the prehospital ECG of chest pain patients. J Electrocardiol. 2023;83(1):34-41 10.1016/j.jelectrocard.2023.10.009 38006762

[51] Tewalker SK, Shukla R. Minimum Skewness based Myocardial Infarction Detection Model using Classification Algorithms. Intern J Electrical Com Eng Systems. 2024;15(9):783-93.

[52] Tharwat A, Gabel T. Parameters optimization of support vector machines for imbalanced data using social ski driver algorithm. Neural Computing and Applications. 2019;1–14.

[53] The MIT-BIH Arrhythmia Database is taken from https://www.kaggle.com/datasets/taejoongyoon/mitbit-arrhythmia-database accessed on July 2025.

[54] Wahid JA, Mingliang X, Ayoub M, Hussain S, Li L, Shi L. A hybrid ResNet-ViT approach to bridge the global and local features for myocardial infarction detection. Scientific Reports. 2024;14(1):4349. 10.1038/s41598-024-54846-8 PMC10883929 38388668

[55] Yadav SS, More SB, Jadhav SM, Sutar SR. Convolutional neural networks based diagnosis of myocardial infarction in electrocardiograms. In Proceedings of 2021 International Conference on Computing, Communication, and Intelligent Systems (ICCCIS), IEEE. 2021;581–586.

[56] Zhou C, Xiao Y, Li L, Liu Y, Zhu F, Zhou W, Yi X, Zhao M. Radiomics Nomogram Derived from Gated Myocardial Perfusion SPECT for Identifying Ischemic Cardiomyopathy. J Imaging Informat Med. 2024;1–10. 10.1007/s10278-024-01145-3 PMC11612043 38806952

